# MHC class I on target cells regulates CD4^+^ T cell-mediated immunity

**DOI:** 10.1038/s41590-026-02480-z

**Published:** 2026-03-24

**Authors:** Emma Lauder, Mahnoor Gondal, Meng-Chih Wu, Akira Yamamoto, Laure Maneix, Dongchang Zhao, Yaping Sun, Marcin Cieslik, Arul M. Chinnaiyan, Pavan Reddy

**Affiliations:** 1 Immunology Program, University of Michigan, Ann Arbor, MI USA; 2 Dan L Duncan Comprehensive Cancer Center, Baylor College of Medicine, Houston, TX USA; 3 Department of Computational Medicine and Bioinformatics, University of Michigan, Ann Arbor, MI USA; 4 Michigan Center for Translational Pathology, University of Michigan, Ann Arbor, MI USA; 5 Program in Immunology and Microbiology, Baylor College of Medicine, Houston, TX USA; 6 Department of Pathology, University of Michigan, Ann Arbor, MI USA; 7 Rogel Cancer Center, University of Michigan, Ann Arbor, MI USA; 8 Howard Hughes Medical Institute, Ann Arbor, MI USA; 9 Department of Urology, University of Michigan Medical School, Ann Arbor, MI USA

**Keywords:** Bone marrow transplantation, MHC class I, MHC class II, Immunosurveillance, Cell death and immune response

## Abstract

Major histocompatibility complex (MHC) class I and class II molecules present antigens to CD8^+^ and CD4^+^ T cells respectively. Here we uncover a previously unrecognized role for MHC class I in modulating CD4^+^ T cell-mediated immunity. In allogeneic graft-versus-host disease and tumor models, we demonstrate that the absence of MHC class I on target cells significantly increases their susceptibility to CD4^+^ T cell cytotoxicity. Transcriptomic and functional studies suggest that this was because of heightened sensitivity to enhanced ferroptosis of the target cells. In large human transcriptomic and sequencing datasets, a role for CD4^+^ T cells in enhancing immune checkpoint blocker-mediated responses in persons with melanoma and mismatch-repair-deficient colon cancers that have downregulated MHC class I was suggested. These findings revise and expand the known role of MHC class I in CD8^+^ T cell and natural killer cell immunity and demonstrate a previously unrecognized role in CD4^+^ T cell-mediated cancer and alloimmunity.

## Abstract (web summary)

Here, the authors suggest that, when major histocompatibility complex class I is downregulated on allogenic or tumor cells, they are more susceptible to CD4^+^ T cell-mediated ferroptosis.

## Main

First studied in the context of allogeneic transplantation, the major histocompatibility complex (MHC) is central to adaptive immune responses. MHC presents antigenic peptides on the cell surface for T cell recognition. This process shapes T cell ontogeny and function by following a class-restricted dichotomy where MHC class I (MHC I) is restricted to driving CD8^+^ T cell immunity and MHC II to CD4^+^ T cell immunity^1^.

While MHC II is restricted to antigen-presenting cells (APCs), MHC I is ubiquitously expressed on all nucleated cells for primary surveillance. Consequently, viruses and tumors frequently downregulate MHC I to evade immune surveillance. Beyond antigen presentation, MHC I regulates CD8^+^ T cell and natural killer (NK) cell function^2^, as well as nonimmune processes, including neuronal pruning, placental development, iron homeostasis and overload (hemochromatosis)^3–5^.

However, given that MHC I is expressed by all nucleated cells, whether MHC I has a role independent of antigen presentation to CD8^+^ T cells, specifically in direct CD4^+^ T cell-mediated target cell lysis, is not known. Herein, we investigated whether the presence of MHC I on a target cell modifies the activation of CD4^+^ T cells and/or the sensitivity and responsiveness of the target cells exclusively to CD4^+^ T cells.

Furthermore, while mutations in the *HFE* gene have a role in iron biology and hereditary hemochromatosis^6^, whether MHC I loss of function leads to enhanced iron-dependent cell death, known as ferroptosis, remains unknown. Recent data have demonstrated a key role for ferroptosis in CD4^+^-mediated and CD8^+^-mediated infection and tumor immunity^7^. However, its role in alloimmunity (allogeneic immune response) remains unexplored. While iron overload has been linked to ferroptosis^8,9^, it is unknown whether MHC I expression, regardless of iron overload, regulates susceptibility to ferroptosis.

Using multiple well-characterized and clinically relevant models of allogeneic graft-versus-host disease (GVHD) and immunologically stringent models of tumor immunity, we explored whether MHC I expression on target cells regulated CD4^+^ T cell-mediated immunity and target tissue ferroptosis. Contrary to the existing paradigm that MHC I only has a role in CD8^+^-mediated or NK cell-mediated immunity, our data demonstrate a heretofore unrecognized role for MHC I in regulating CD4^+^ T cell-mediated immune responses through induction of target cell ferroptosis.

## Results

### MHC I expression regulates CD4^+^ T cell-mediated immunopathology

**Fig. 1:**
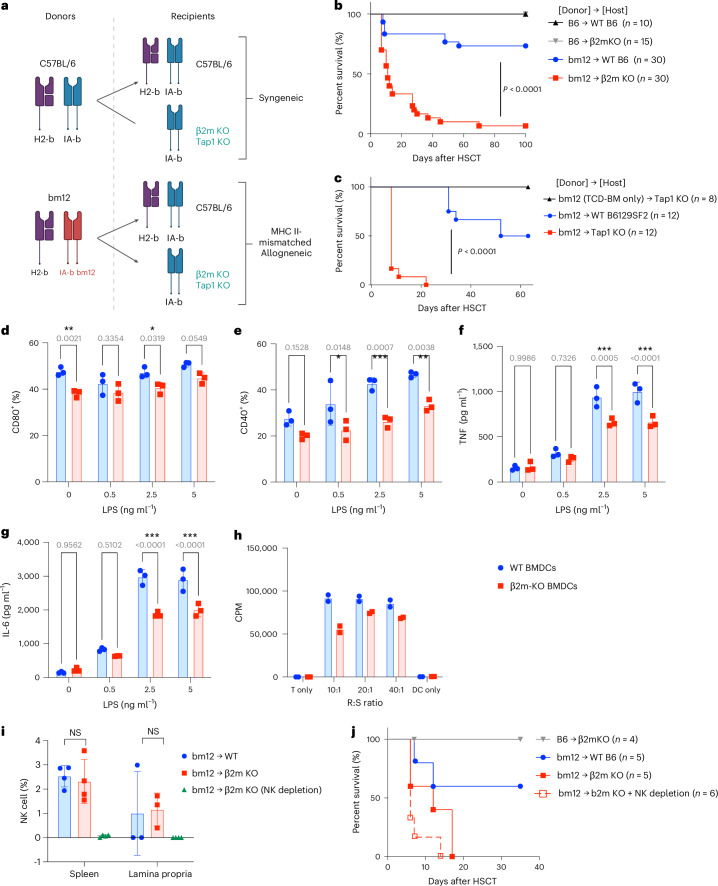
MHC I-deficient mice are more susceptible to CD4^+^ T cell attack. **a**–**c**, WT B6, β2m-KO and Tap1-KO mice received 10 Gy of TBI followed by transplantation of 2 × 10^6^–2.5 × 10^6^ T cells and 5 × 10^6^ BM cells from either syngeneic B6 WT or MHC II-mismatched bm12 donors (**a**). Survival (**b**, *P* = 7.4 × 10^−8^; **c**, *P* = 1.7 × 10^−5^) was monitored after HSCT. **d**–**g**, BMDCs from WT and β2m-KO mice were stimulated with LPS for 16 h. The surface expression of costimulatory molecules (**d**,**e**) and cytokine secretion (**f**,**g**) were measured (**f**, *P* = 6.2 × 10^−5^; **g**, *P* = 1 × 10^−6^ and 1.5 × 10^−5^). Data are the mean ± s.d. of *n* = 3 technical replicates from one representative experiment of three independent biological replicates. **h**, bm12 T cell proliferation was measured with ^3^H-thymidine 16 h after coculture with LPS-stimulated WT or β2m-KO BMDCs. Data are the mean ± s.d. of *n* = 2 technical replicates from one representative experiment of three independent biological replicates. **i**, NK cell frequency was measured by flow cytometry in the spleen and intestinal lamina propria of allogeneic recipients 5 days after HSCT. Recipients were pretreated 1 day before transplantation with either anti-NK1.1 antibody or IgG isotype control. Data are the mean ± s.d. of *n* = 3–4 biological replicates. NS, not significant. **j**, Survival of WT B6 and β2m-KO mice receiving either anti-NK1.1 antibody or IgG isotype control was monitored after HSCT. Statistical significance was determined using a log-rank (Mantel–Cox) test (**b**,**c**,**j**), two-way analysis of variance (ANOVA) with Šidák’s post hoc test (**d**–**g**) or one-way ANOVA with Tukey’s post hoc test (**i**).

To isolate the role of MHC I in an exclusively CD4^+^ T cell-mediated cytopathic reactions against nontransformed cells, we used an MHC II-disparate, MHC I-matched model of gastrointestinal (GI)-GVHD (Fig. 1a) by transplanting T cell-depleted bone marrow (TCD-BM) and purified splenic T cells from allogeneic bm12 (*H2*-*Ab1*^*bm12*^) or syngeneic B6 (*H2-Ab1*^*b*^) donors into B6 recipients. To model MHC I deficiency, we used B6 β2-microglobulin-knockout (β2m-KO) mice, which lack MHC I expression across all tissues, as recipients compared to wild-type (WT) B6. This ensures a consistent alloreactive donor CD4^+^ T cells, with no confounding effects on donor CD8^+^ T cell antigen recognition and activation, regardless of host MHC I expression. Syngeneic recipients served as controls for any irradiation or lymphopenia-induced effects. Thus, the primary variable for any differences in GVHD between allogeneic B6 WT and β2m-KO recipients is solely from the absence of β2m in target tissues and their susceptibility to CD4^+^ T cell-mediated alloreactivity.

**Extended Data Fig. 1:**
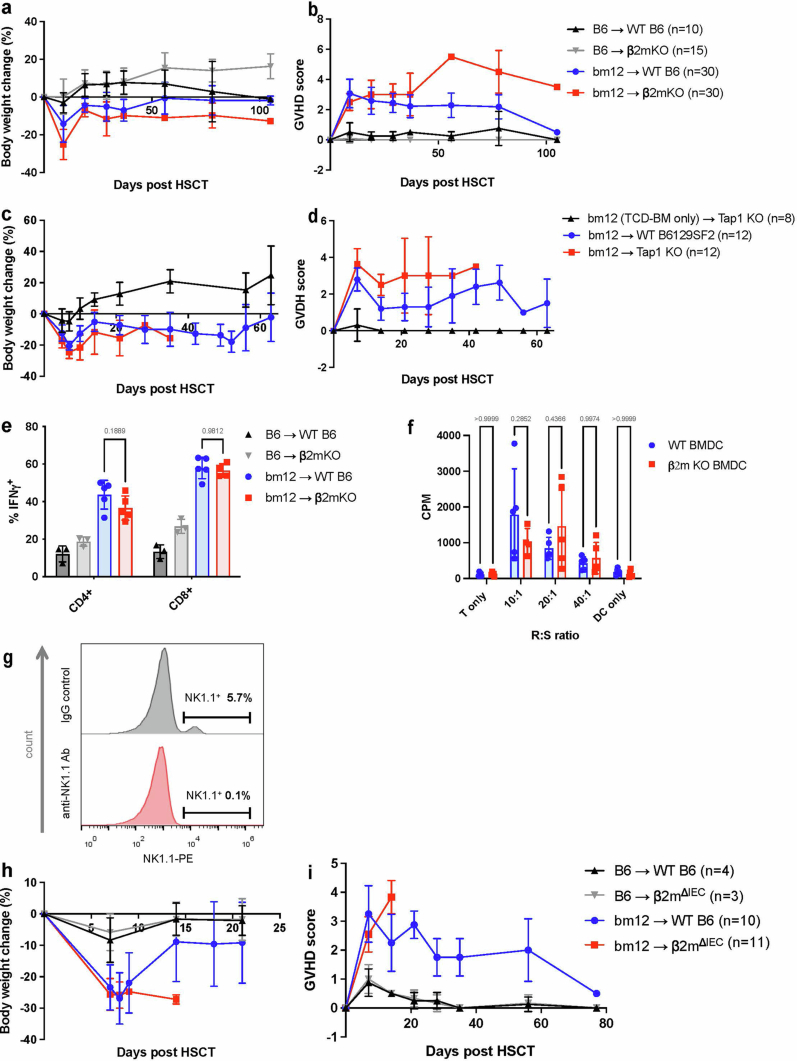
Body weight changes and clinical GVHD scores in CD4^+^-mediated GVHD model. **a-d,h,i**, WT B6, β2 m KO, Tap1KO, and β2 m^∆IEC^ mice received 10 Gy TBI followed by transplantation of T cells and bone marrow cells from either syngeneic B6 WT or MHC II-mismatched bm12 donors. Body weight change (**a,c,h**) and GVHD score (**b,d,i**) were recorded. **e**, Splenocytes were isolated from recipient mice four days after transplantation and IFNγ was measured in CD4^+^ and CD8^+^ T cells. Data are mean ± SD of n = 3 (syngeneic) or 5 (allogeneic) biological replicates. **f**, bm12 T cell proliferation was measured after co-culture with unstimulated WT or β2 m KO BMDCs. Data are mean ± SD of n = 5 technical replicates from one representative experiment of three independent biological replicates. **g**, NK cell frequency was quantified in the CD45^+^CD3^−^ population from the spleens of mice that received either an anti-NK1.1 antibody or an IgG isotype control 18 days prior to analysis. Two-way ANOVA with Tukey’s post-hoc test (**e**) or Sidak’s post-hoc test (**f**) were used to determine significance.

Following hematopoietic stem cell transplantation (HSCT), while the allogeneic WT recipients demonstrated clinical GVHD and mortality at the expected severity, the allogeneic β2m-KO recipients demonstrated significantly greater GVHD severity and mortality when compared to WT controls (Fig. 1b and Extended Data Fig. 1a,b). By contrast, all of the syngeneic WT and β2m-KO recipients survived with no GVHD, demonstrating that the increase in mortality is driven only by the alloreactive bm12 CD4^+^ T cells.

To ensure this effect resulted from MHC I deficiency rather than β2m-specific effect, we next used transporter associated with the antigen processing 1 (Tap1)-KO mice as a allogeneic recipients using the MCH II HSCT as above. To further confirm a role for allogeneic CD4^+^ T cells in mediating GVHD responses, we used allogeneic TCD-BM instead of syngeneic donors as non-GVHD controls. Consistent with observations in the β2m-KO model, Tap1-KO allogeneic recipients receiving CD4^+^ T cells and TCD-BM but not TCD-BM alone demonstrated heightened GVHD mortality and severity (Fig. 1c and Extended Data Fig. 1c,d). These findings suggest that the susceptibility to heightened CD4^+^ T cell-mediated GVHD severity is a general feature of MHC I deficiency in the hosts.

### Host tissue deficiency of MHC I does not alter the activation of alloreactive CD4^+^ T cells

We next examined whether the increased mortality in β2m-KO mice was secondary to factors other than a target organ-specific sensitivity to CD4^+^ T cell-mediated cytotoxicity. Specifically, first, we assessed absence of MHC I on target cell affected donor CD4^+^ T cell activation after HSCT. Compared to WT control, allogeneic β2m-KO recipients showed comparable percentages of donor CD4^+^IFNγ^+^ or CD8^+^IFNγ^+^ cells (Extended Data Fig. 1e). Furthermore, β2m-KO BM-derived dendritic cells (BMDCs) demonstrated lesser expression of costimulatory molecules (CD80 and CD40), IL-6 and TNF secretion, and ability to stimulate bm12 CD4^+^ T cell proliferation compared than WT B6 BMDCs (Fig. 1d–h). Notably, unstimulated WT and β2m-KO BMDCs had similar T cell stimulation potential (Extended Data Fig. 1f). These data demonstrate that the increased mortality in β2m-KO mice is not driven by greater activation of alloreactive T cells or augmented host APC function.

Next, we explored the role of donor NK cells as the absence of MHC I on host targets may drive the donor NK cell-mediated killing^10^. We stringently depleted NK cells from both the graft and the recipients by administering an anti-NK1.1 antibody to both donors and the hosts 1 day before transplantation. The efficacy of a single 200-µg dose of anti-NK1.1 antibody was sufficient to deplete NK cells from the spleen for at least 18 days after the administration of antibody (Extended Data Fig. 1g). We also confirmed the NK depletion in recipient mice 5 days after HSCT in the spleen and intestinal lamina propria of the hosts (Fig. 1i). Despite NK depletion, the β2m-KO allogeneic recipient mice still demonstrated significantly greater GVHD mortality (Fig. 1j).

### Target cell-specific deficiency of MHC I makes them uniquely susceptible to CD4^+^ T cells

**Extended Data Fig. 2:**
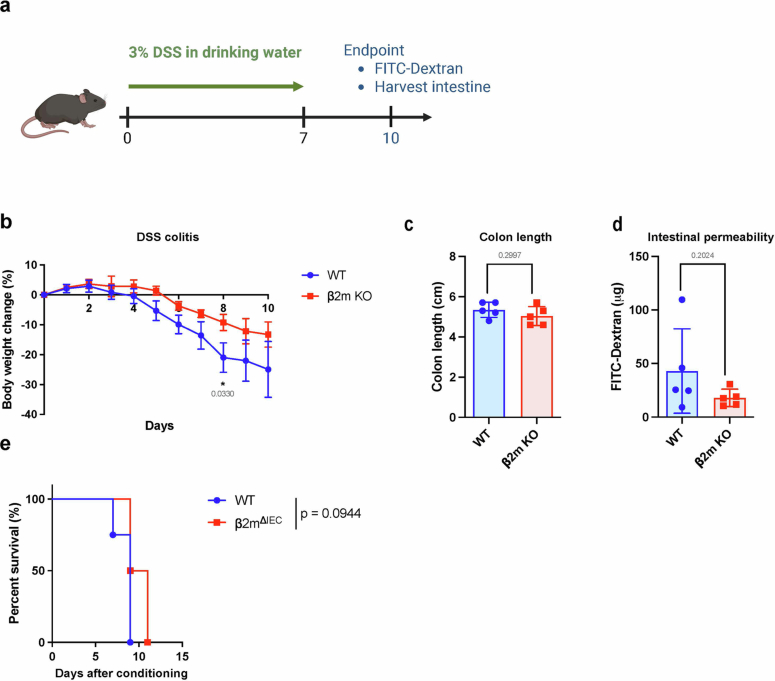
Response of β2 m KO mice to non-CD4^+^ T cell-mediated inflammation. **a-d**, Colitis was induced via DSS administration in WT and β2 m KO mice (**a**). Bodyweight changes were recorded daily (**b**). On day 10, mice were euthanized, and endpoint analyses associated with colitis phenotype were collected: colon length (**c**) and intestinal permeability (**d**). Data are mean ± SD of n = 5 biological replicates. **e**, Survival was monitored after WT (β2 mflVilCre-) or β2m^∆IEC^ (β2mflVilCre + ) mice received 10 Gy TBI (n = 4 in each group). Two-way repeated measures ANOVA with Sidak’s multiple comparisons test (**b**), two-tailed unpaired t-test (**c**-**d**), or Log-rank (Mantel-Cox) test (**e**) were used to determine significance.

To assess whether MHC I deficiency increased the vulnerability of the target cells to any inflammatory damage that is not mediated directly by CD4^+^ T cells, we used the dextran sodium sulfate (DSS) colitis model where the intestinal damage is caused by chemical inflammation (Extended Data Fig. 2a). β2m-KO mice lost less weight than WT controls (Extended Data Fig. 2b) and exhibited no significant difference in colon lengths or in intestinal permeability (Extended Data Fig. 2c,d). Furthermore, lethal irradiation resulted in similar mortality between WT and β2m-KO mice (Extended Data Fig. 2e). These results collectively indicate that β2m-KO mice do not have an increased susceptibility chemical or radiation-induced inflammation.

**Fig. 2:**
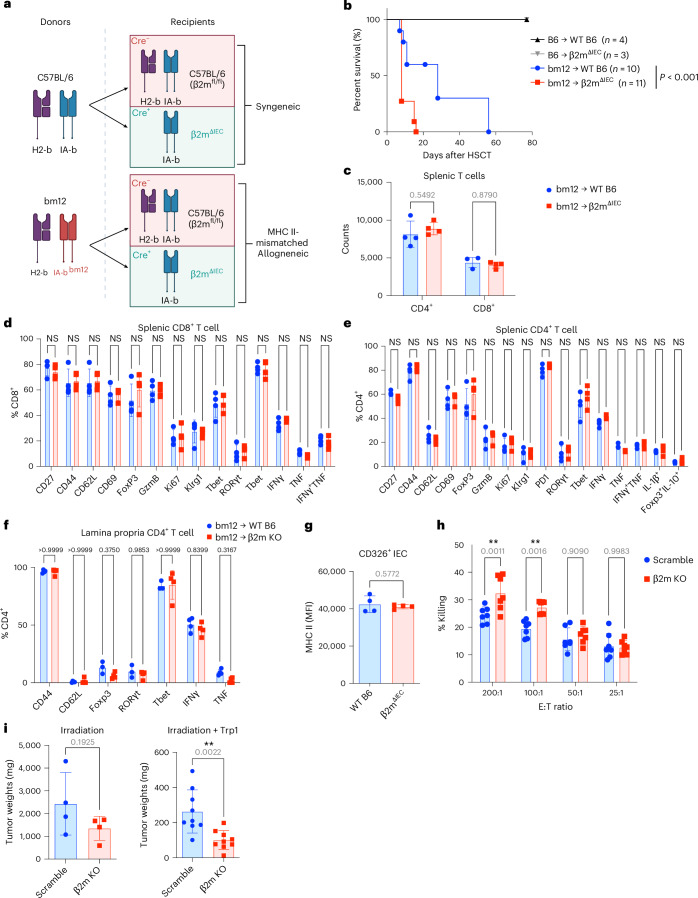
Transformed and GVHD target cells are more susceptible to CD4^+^ T cell-mediated death. **a**,**b**, WT B6 (β2m^fl/fl^*Vil1*-*cre*^−^) and β2m^∆IEC^ mice received 10 Gy of TBI followed by transplantation of 2 × 10^6^–2.5 × 10^6^ T cells and 5 × 10^6^ BM cells from either syngeneic B6 WT or MHC II-mismatched bm12 donors (**a**). Survival (**b**) was monitored after HSCT. **c**–**e**,**g**, Splenic T cells and IECs were isolated from allogeneic WT and β2m^∆IEC^ recipients 7 days after transplantation. Absolute T cell counts (CD4^+^ or CD8^+^) were recorded (**c**) and the percentage of positive cells for activation markers and T cell subsets was recorded in CD8^+^ T cells (**d**) or CD4^+^ T cells (**e**). MHC II surface level was measured in CD326^+^ IECs (**g**). Data are the mean ± s.d. of *n* = 3–4 biological replicates. **f**, Intestinal lamina propria leukocytes were isolated from allogeneic WT or β2m-KO recipients 5 days after transplantation and the percentage of positive cells for activation markers and T cell subsets was recorded in CD4^+^ T cells. Data are the mean ± s.d. of *n* = 3–4 biological replicates. **h**, Cell death (% killing) was measured in β2m-KO or scramble B16 cells after coculture with activated bm12 CD4^+^ T cells for 6 h. T cells (effectors) and B16 cells (targets) were cocultured at varied E:T ratios to assess cell death (**h**). Data are the mean ± s.d. of technical replicates from one representative experiment of three independent biological replicates. **i**, WT C57BL/6 mice are injected s.c. with scramble or β2m-KO B16 cells. Then, 10 days after s.c. injection, mice were sublethally irradiated with 5 Gy. Mice receiving Trp1 T cells were injected i.v. 4 h after irradiation. Then, 10 days after T cell transfer, tumor-bearing mice were killed and tumor weights were measured. Data are the mean ± s.d. of *n* = 3 (irradiation) or 9 (irradiation + Trp1 T cells) biological replicates. Statistical significance was determined using a log-rank (Mantel–Cox) test (**b**), two-way ANOVA with Šidák’s post hoc test (**c**–**f**,**h**), or two-tailed unpaired *t*-test (**g**,**i**).

To determine whether the increased GVHD mortality in MHC I-deficient mice was indeed specifically because of CD4^+^ T cell-mediated target organ-specific and not from the systemic consequence of MHC I deficiency, we generated intestinal epithelial cell (IEC)-specific β2m-KO mouse model (β2m^fl/fl^*Vil1-cre*^+^, hereafter β2m^∆IEC^). Following HSCT (Fig. 2a), allogeneic β2m^∆IEC^ mice demonstrated significantly greater mortality, body weight loss and GVHD score compared to β2m^fl/fl^ (WT) mice (Fig. 2b and Extended Data Fig. 1h,i). Donor T cells in the host spleen after transplant demonstrated similar activation and differentiation between WT and the β2m^∆IEC^ allogeneic recipient mice (Fig. 2c–e). Consistent with the trend observed in the spleen, the activated and IFNγ^+^CD4^+^ T cells in the intestinal lamina propria were comparable between WT and β2m-KO recipients (Fig. 2f). Furthermore, the absence of MHC I on β2m^∆IEC^ IECs did not increase MHC II expression on IECs after transplant (Fig. 2g).

### CD4^+^ T cell-mediated tumor immunity is regulated by MHC I

We next explored the generalizability of MHC I-deficient tissue’s vulnerability to CD4^+^ T cell-mediated cell death to transformed and tumor cells. We generated CRISPR–Cas9-mediated β2m-KO B16 melanoma cells. The KO was validated by western blotting (Extended Data Fig. 7a) and flow cytometry to confirm the loss of H2kb surface expression (Extended Data Fig. 7b). We first performed cytotoxic lymphocyte (CTL) assay by coculturing scramble or β2m-KO B16 cells with activated alloreactive MHC II-disparate bm12 T cells and measuring cell killing through a CTL chromium release assay. β2m-KO B16 cells showed significantly greater cell death at higher effector-to-target (E:T) ratios when compared to control scramble B16 cells (Fig. 2h).

We next examined whether this was valid in vivo using a CD4^+^ T cell-mediated tumor immunity model. Scramble or β2m-KO B16 tumor cells were injected subcutaneously (s.c.) into syngeneic immune-ablated B6 mice, followed by adoptive transfer of MHC II-restricted Trp1 CD4^+^ T cells recognizing the Tyrp1 melanoma antigen^11–13^. While growth kinetics were similar in the absence of Trp1 T cells, β2m-KO B16 tumors showed significantly greater regression upon Trp1 T cell transfer (Fig. 2i). Collectively, these data demonstrate that the absence of β2m increases the susceptibility of tumor cells to MHC II-restricted CD4^+^ T cell-mediated cytotoxicity.

### Mechanisms of enhanced sensitivity of MHC I-deficient targets to CD4^+^ T cells

**Fig. 3:**
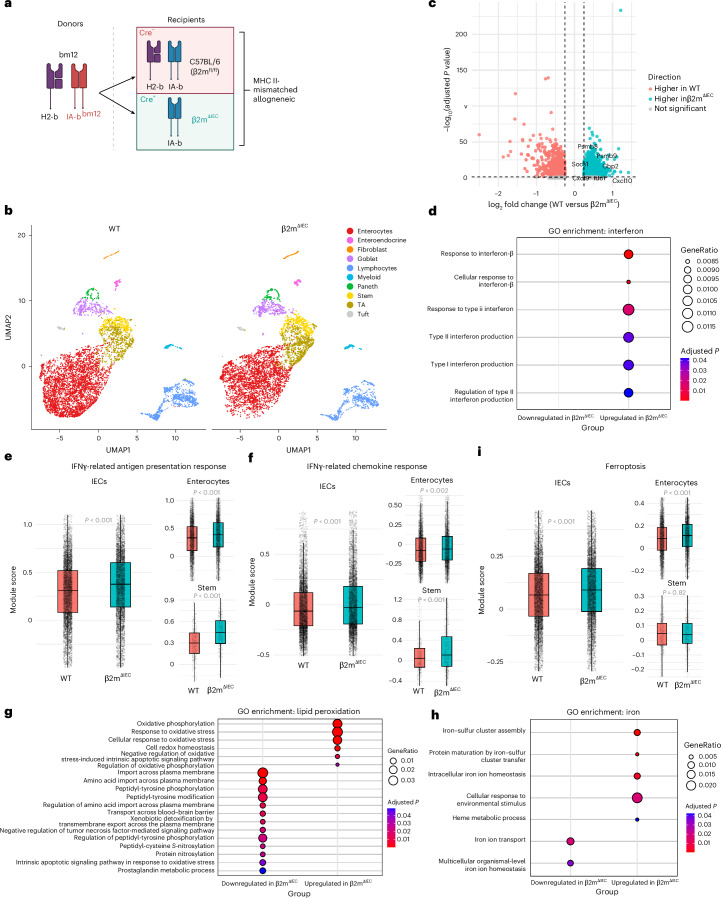
scRNAseq analysis of IECs after allogeneic HSCT in Cre^−^ (WT) and Cre^+^ (β2m^∆IEC^) mice. WT B6 and β2m^∆IEC^ mice received 10 Gy of TBI followed by transplantation of T cells and BM cells MHC II-mismatched bm12 donors. Then, 6 days after transplantation, IECs were enriched and a single-cell library was made (**a**). Clusters from single-cell analysis (**b**) were made. Differential expression analysis was performed on IECs. IFNγ-related genes were subsequently extracted for display. Differential expression was assessed using a two-sided Wilcoxon rank-sum test (Seurat FindMarkers), with multiple-testing correction performed using the Benjamini–Hochberg method. An adjusted *P* < 0.05 was considered statistically significant. (**c**). **d**,**g**–**h**, Gene Ontology term overenrichment analysis was performed on IECs and pathways related to the interferon response signature, lipid peroxidation and iron are shown. Gene Ontology (biological process) overrepresentation analysis was performed using clusterProfiler (enrichGO), with Benjamini–Hochberg correction for multiple testing. An adjusted *P* < 0.05 was considered significant. **e**,**f**,**i**, Module scores for IFNγ-related antigen presentation and chemokine genes were calculated and compared across all IECs, as well as specifically in enterocytes and stem cells (**e**, IECs, *P* = 3.3 × 10^−24^; enterocytes, *P* = 1.3 × 10^−13^; stem cells, *P* = 1.76 × 10^−17^. **f**, IECs, *P* = 1.77 × 10^−15^; enterocytes, *P* = 0.002; stem cells, *P* = 1.1 × 10^−4^). Module scores for ferroptosis-related pathways in all IECs, as well as specifically in enterocytes and stem cells (**i**). Box plots show the median (center line), 25th–75th percentiles (box limits) and minimum and maximum values within 1.5× the interquartile range (IQR) (whiskers); each dot represents an individual cell pooled from four biological replicates. For visualization purposes, the *y* axis was restricted to the 2.5th–97.5th percentile range of module scores and no data points were excluded from the analysis. Statistical significance was calculated using a two-sided Welch’s *t*-test, with *P* values indicated for each comparison.

To uncover the potential mechanisms driving the greater sensitivity of MHC I-deficient target cells to CD4^+^ T cell-mediated cytotoxicity, we performed single-cell RNA sequencing (scRNA-seq) on the IECs obtained from allogeneic β2m^∆IEC^ or WT recipients as described before^14^ (Fig. 3a). While initial clustering showed similar IEC subtypes and immune subsets distributions between the groups (Fig. 3b), differential expression analysis on IECs identified a significantly enriched IFNγ response signature in the β2m^ΔIEC^ group (Fig. 3c,d). Specifically, IECs in β2m^ΔIEC^ group exhibited higher module scores for genes representing key IFNγ-driven programs (Fig. 3e,f). Given the established role of IFNγ in inducing downstream oxidative stress, we further examined related pathways and found that the β2m^ΔIEC^ group exhibited significant enrichment in lipid peroxidation (Fig. 3g), alongside altered iron metabolism signatures (Fig. 3h).

**Extended Data Fig. 3:**
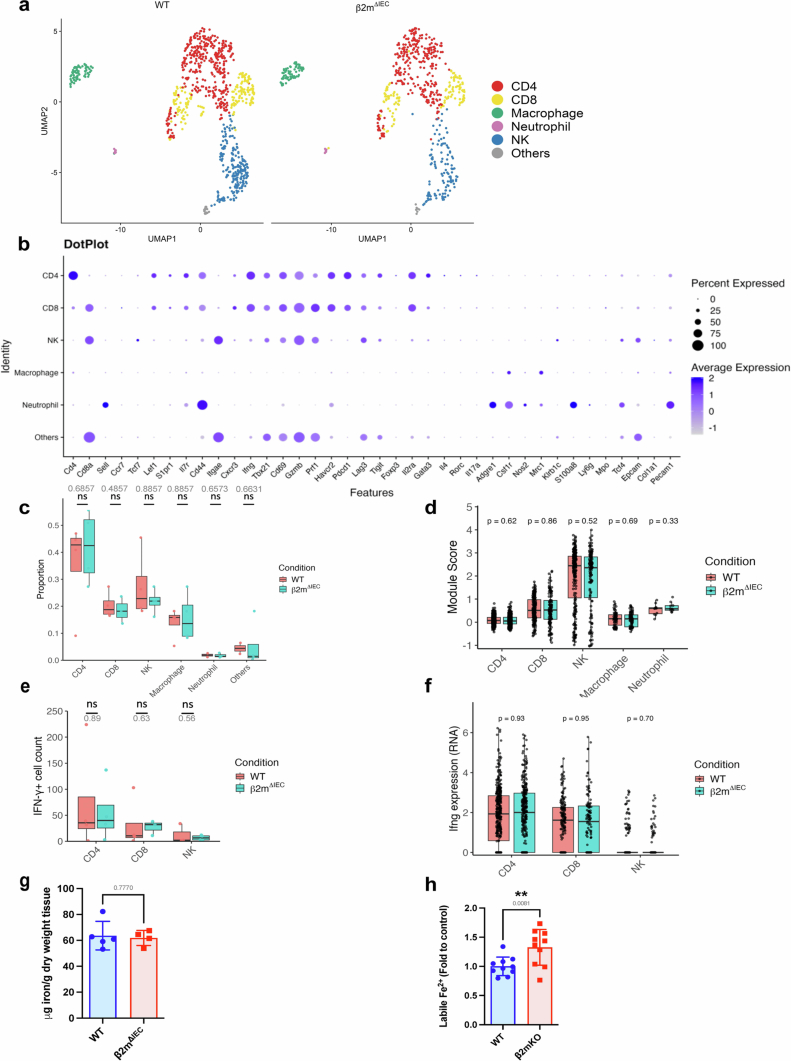
Single Cell RNAseq Analysis of immune cells post- HSCT in Cre^−^ (WT) and Cre^+^ (β2 m^∆IEC^) mice. **a-f**, Subclustering was performed on the immune cell population identified in the initial clustering to examine in greater detail the distribution and functions of immune cell subsets (**a**). Dot plot showing the expression of canonical marker genes across immune cell clusters identified in the scRNA-seq dataset (**b**). Comparison of cell proportions, activation marker expression, number of IFNγ-positive cells, and interferon-γ expression levels in each cell type was performed (**c-f**). Box plots show the median (center line), 25th–75th percentiles (box limits), and min/max values within 1.5 × IQR (whiskers) with each dot represents either a pseudobulk aggregate from one biological replicate (**c**, **e**) or an individual cell pooled from n = 4 independent mice (**d**, **f**). **g**, Iron content was measured in the small intestine via ICP-OES. Data are mean ± SD of n = 4-5 biological replicates. **h**, Labile Fe^2+^ was measure via flow cytometry in the IECs isolated from allogeneic WT or β2 m KO recipients six to seven days after HSCT. Data are mean ± SD of n = 10 biological replicates. Two-sided Wilcoxon rank-sum test wtih multiple comparison correction (Benjamini-Hochberg) (**c**-**f**) or two-tailed unpaired t test (**g**, **h**) were used to determine significance.

Subclustering of myeloid and lymphocyte populations (Extended Data Fig. 3a,b) revealed no significant differences in cell proportions (Extended Data Fig. 3c) or activation marker module scores between the two groups across immune cell types (Extended Data Fig. 3d). Additionally, the frequency and expression levels of IFNγ-producing immune cells were comparable between groups (Extended Data Fig. 3e,f). These finding, consistent with our flow cytometry data (Fig. 2c–f), collectively suggest that changes in immune cell subsets or their activation were similar between the groups and, thus, not the driving factor in causing the pathway alternation within β2m^ΔIEC^ IECs.

Given this similar immune environment and because the pathway analysis showed alterations in iron and lipid metabolism pathways, we next hypothesized that ferroptosis, an iron-dependent cell death characterized by the accumulation of lipid peroxides, could be causing increased cell death in MHC I-deficient target cells. In accordance with our hypothesis, focused pathway analysis revealed a significant enrichment of ferroptosis in β2m^ΔIEC^ IECs (Fig. 3i). Next, to confirm whether an iron overload in the intestine was the cause of the increased ferroptotic cell death in β2m^ΔIEC^ mice, we measured iron content in the intestine. While total intestinal iron content (Fe^2+^ and Fe^3+^) remained similar (Extended Data Fig. 3g), Fe^2+^ levels were elevated in allogeneic β2m^ΔIEC^ recipients compared to WT. (Extended Data Fig. 3h). These data suggested a proferroptotic response, characterized by Fe^2+^ accumulation, as a putative mechanism of increased target cell death in the absence of MHC I.

### Ferroptosis contributes to the sensitivity of MHC I-deficient target cells to CD4^+^ T cell-mediated immunity

**Fig. 4:**
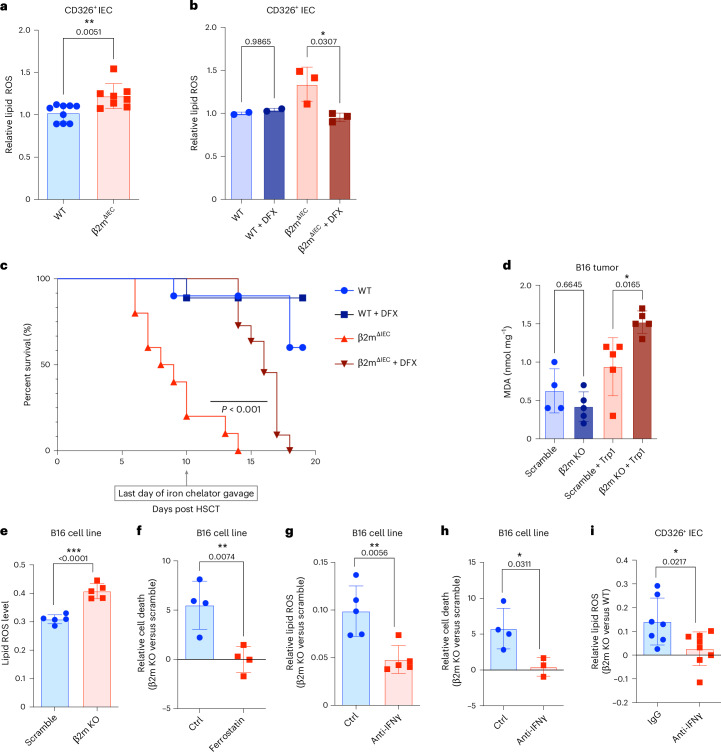
Ferroptosis in target cells of GVHD. **a**,**b**,**d**,**i**, Lipid peroxidation was measured by C11 BODIPY 581/591 staining in GVHD target cells, IECs, 7 days after transplant (**a**,**b**,**i**) or measured using MDA in tumor cells after radiation with or without Trp1 T cell transfer (**d**). Data are the mean ± s.d. of individual biological replicates. **c**, Mice received 10 Gy of TBI 1 day before HSCT. The iron chelator, DFX, was administered from 5 days before transplant to 10 days after transplant in mice receiving a bm12 HSCT. Survival was recorded after HSCT (*P* = 6.6 × 10^−6^). **e**–**h**, Lipid peroxidation measured by C11 BODIPY 581/591 staining (**e**,**g**) and cell death (**f**,**h**) measured in β2m-KO or scramble B16 cells after coculture with activated bm12 CD4^+^ T cells with a ratio of 1:5 for 6 h, with or without 10 µM ferrostatin or 10 µg ml^−1^ anti-IFNγ antibody (**e**, *P* = 9.6 × 10^−5^). Data are the mean ± s.d. of technical replicates from one representative experiment of three independent experiments. Relative lipid peroxidation (**g**,**i**) or relative cell death (**f**,**h**) was calculated as the difference in lipid reactive oxygen species levels or cell death between β2m-KO and WT mice under the same treatment. Statistical significance was determined using a two-tailed unpaired *t*-test (**a**,**e**–**i**), log-rank (Mantel–Cox) test (**c**) or one-way ANOVA with Tukey’s post hoc test (**b**,**d**).

**Extended Data Fig. 4:**
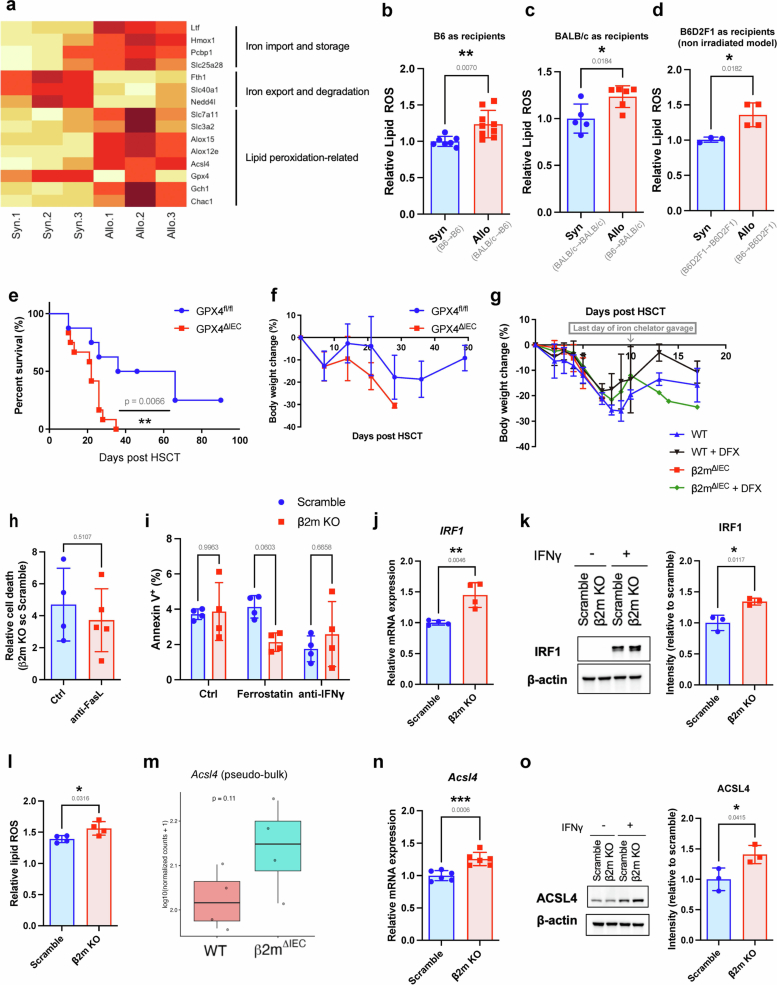
Molecular mechanisms promoting ferroptosis in MHC I-deficient target cells. **a**, Heatmap of gene expression in allogeneic and syngeneic IECs including iron metabolism and lipid peroxidation. **b-d**, Lipid peroxidation was measured with C11 BODIPY 581/591 staining in IECs isolated from syngeneic (Syn) and allogeneic (Allo) recipients in a major MHC-mismatched model (**b-c**) and a non-irradiated model (**d**). Data are mean ± SD with each point represents biological replicates. **e-f**, Survival (**e**) and body weight loss (**f**) was monitored after allogeneic transplant in GPX4^∆IEC^ and GPX4^fl/fl^ mice. **g**, Body weight loss of allogenic WT or β2m^∆IEC^ receiving with or without iron chelators, DFX, was monitored after HSCT. **h-i**, β2m KO or scramble B16 cells were co-cultured with activated bm12 CD4^+^ T cells for 6 hours, with or without ferrostatin or neutralizing antibodies. After co-culture, cell death (**h**) and apoptosis (**i**) were measured. Data are mean ± SD with each point represents technical replicates from one representative experiment of three independent experiments. **j,k,n,o**, IRF1 and ACSL4 mRNA and protein levels were measured 6 h (**j**) or 24 h (**k, n, o**) after IFNγ stimulation and normalized to scramble group. Bar graph shows densitometric quantification of Western blot bands from three independent experiments (**k, o**). Data are mean ± SD with each point represents the independent experiments. **l**, Scramble and β2 m KO B16 cells were cultured for 40hrs with IFNγ and 8 hr incubation with RSL3, and lipid peroxidation were measured. Relative lipid ROS was calculated as the fold change relative to the untreated control. Data are mean ± SD with each point represents technical replicates from one representative experiment of three independent experiments. **m**, Normalized Acsl4 expression in IECs based on pseudobulk analysis was compared between WT and β2m^ΔIEC^ mice (m). Box plots show the median (center line), 25th–75th percentiles (box limits), and min/max values within 1.5 × IQR (whiskers) with each dot represents either a pseudobulk aggregate from one biological replicate. Two-tailed unpaired t-test (**b**-**d**, **i**-**l**, **n**-**o**), Log-rank (Mantel-Cox) test (**e**), two-way ANOVA with Sidak’s posthoc test (**h**), or two-sided Wilcoxon rank-sum test (**m**) were used to determine significance. Source data

Next we directly assessed for ferroptosis of the IECs to corroborate scRNA-seq data. Consistent with it, allogeneic β2m^∆IEC^ IECs exhibited significantly higher lipid peroxidation than WT controls (Fig. 4a), suggesting that β2m^∆IEC^ IECs are more proferroptotic. Given that the role of ferroptosis in GI-GVHD is not well established, we next investigated whether ferroptosis indeed contributed to GI-GVHD-related mortality. Comparison of the gene expression programs in IECs from WT allogeneic and syngeneic recipients demonstrated significant alternations in pathways related to iron metabolism and lipid peroxidation (Extended Data Fig. 4a). In alignment with expression data, IECs from allogeneic recipients demonstrated increased lipid peroxidation in multiple irradiated (Extended Data Fig. 4b,c) or nonirradiated GI-GVHD models (Extended Data Fig. 4d) when compared to syngeneic controls. Furthermore, IEC-specific KO of major ferroptosis inhibitor glutathione peroxidase 4 (GPX4^∆IEC^) exacerbated GVHD mortality and severity when compared to allogeneic corn-oil-treated controls (GPX4^fl/fl^) (Extended Data Fig. 4e,f), demonstrating ferroptosis contributes to GI-GVHD.

Having established that ferroptosis contributes to GVHD pathology, we next sought to confirm that iron-dependent ferroptosis is the critical contributor to the increased cell death in MHC I-deficient recipients when compared to WT allogeneic recipients. To test this, we administered the iron chelator deferasirox (DFX) to allogeneic β2m^∆IEC^ recipients. The administration of DFX significantly reduced lipid peroxidation (Fig. 4b), improved survival and body weight and reduced clinical severity of GVHD in allogeneic β2m^∆IEC^ recipients when compared to diluent-treated β2m^∆IEC^ mice (Fig. 4c and Extended Data Fig. 4g), suggesting that a heightened vulnerability of MHC I-deficient intestinal cells to GI-GVHD.

To assess the generalizability of this mechanism, we used the B16 tumor model. In vivo, β2m-KO B16 tumors exhibited significantly higher lipid peroxidation compared to both scramble B16 control tumors and those that did not receive Trp1 T cells (Fig. 4d). Consistently, in the in vitro CTL assay, β2m-KO cells demonstrated a higher level of lipid peroxidation compared to scramble controls (Fig. 4e). The heightened cell death in β2m-KO cells was decreased by ferroptosis inhibitor, ferrostatin (Fig. 4f), but not by anti-FasL (Extended Data Fig. 4h). Furthermore, annexin V staining showed no differences in apoptosis between groups (Extended Data Fig. 4i). These suggest that the ferroptosis directly contributed to the enhanced cell death in β2m-KO cells.

Next, because scRNA-seq pathway analysis demonstrated an enriched IFNγ response signature along with perturbations in lipid peroxidation pathways in the MHC I-deficient target cells, we first confirmed the upregulation of IFNγ signaling in MHC I-deficient cells to validate the mechanistic link between enhanced IFNγ signaling and enhanced ferroptosis. Upon IFNγ stimulation, β2m-KO B16 cells exhibited higher IRF1 mRNA and protein levels, indicating an enhanced sensitivity to IFNγ-mediated signaling (Extended Data Fig. 4j–k). When compared to WT, β2m-KO cells showed a greater lipid peroxidation when treated with IFNγ and the ferroptosis inducer RSL3 (Extended Data Fig. 4l), whereas anti-IFNγ antibody treatment decreased lipid peroxidation and abolished the difference in cell death between β2m-KO and scramble control in the in vitro CTL assay (Fig. 4g,h). Similarly, in vivo administration of anti-IFNγ antibody abolished the difference of lipid peroxidation in IECs between WT and β2m-KO allogeneic recipients (Fig. 4i).

To further characterize the molecular link between increased IFNγ signaling signature and ferroptosis, we hypothesized that β2m-deficient targets will show an augmentation of the IFNγ–IRF1–ACSL4 axis, as ACSL4 can promote lipid accumulation and ferroptosis^15^. Consistent with this notion, the in vivo scRNA-seq data revealed a trend toward higher *Acsl4* mRNA levels in allogeneic β2m^ΔIEC^ mice (Extended Data Fig. 4m). Importantly, consistent with this, we observed an increase in ACSL4 expression at both transcript and protein levels in β2m-KO cells upon IFNγ stimulation (Extended Data Fig. 4n,o). Collectively, these data suggest that IFNγ mediated inflammation derived from CD4^+^ T cells drives enhanced ferroptosis in β2m-deficient target cells.

### CD4^+^ T cell infiltration correlates with responses in MHC I-downregulated cancers

**Fig. 5:**
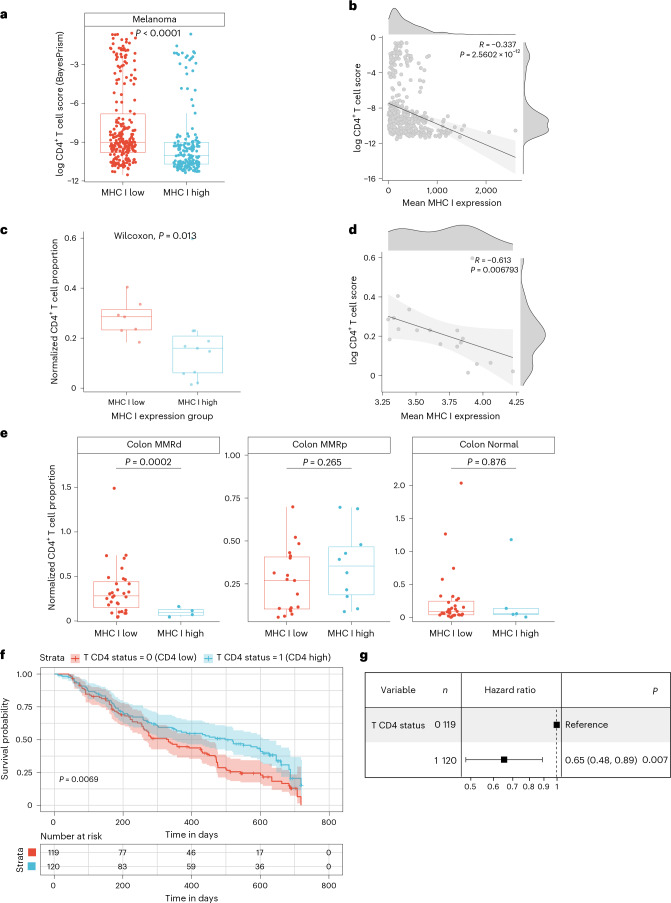
CD4^+^ T cell abundance in relation to MHC I expression across bulk and single-cell datasets. **a**,**b**, Analysis of melanoma bulk RNA-seq datasets from TIGER database. **a**, The log-transformed CD4^+^ T cell scores (as measured by BayesPrism estimates) in MHC I-high (*n* = 165) and MHC I-low (*n* = 249) tumors (two-sided unpaired *t*-test, *P* = 3.76 × 10^−6^). **b**, Spearman correlation (*ρ*) between mean MHC I expression (average of HLA-A, HLA-B and HLA-C) and log-transformed CD4^+^ T cell scores (two-sided Spearman rank correlation, *ρ* = −0.337, *P* = 2.56 × 10^−12^). **c**,**d**, Analysis of single-cell ICB datasets derived from melanoma and basal cell carcinoma samples. **c**, Normalized CD4^+^ T cell proportions in MHC I-high (*n* = 11) and MHC I-low (*n* = 7) tumors (two-sided Wilcoxon rank-sum test, *P* = 0.0013). **d**, Spearman correlation between mean MHC I expression and CD4^+^ T cell scores (two‑sided Spearman rank correlation, *ρ* = −0.613, *P* = 0.00679). **e**, Normalized CD4^+^ T cell proportions in colon cancer scRNA-seq datasets across MMRd (*n* = 35), MMRp (*n* = 29) and normal (*n* = 36) samples in MHC I-high and MHC I-low tumors (two-sided unpaired *t*-test: MMRd, *P* = 0.000222; MMRp, *P* = 0.265; normal, *P* = 0.876). **f**, Kaplan–Meier survival curves comparing overall survival between participants with high versus low CD4^+^ T cell abundance, with 95% confidence intervals shown as error bars. A two-sided log-rank test was used (*P* = 0.0069). **g**, Forest plot of a univariate Cox proportional hazards model for overall survival using CD4^+^ T cell abundance as a binary predictor (high versus low; *n* = 239 participants), reporting the hazard ratio (0.65) and two-sided *P* value (0.007). The point represents the hazard ratio and the horizontal line shows the 95% confidence interval. Box plots show the median (center line), 25th–75th percentiles (box limits) and minimum and maximum values within 1.5× the IQR (whiskers). Linear regression lines in **b**,**d** indicate least-squares fits with 95% confidence intervals. Each point represents an independent tumor sample.

To assess the clinical relevance of our findings, we first analyzed bulk RNA-seq datasets from the TIGER database of persons with melanoma treated with immune checkpoint inhibitors (ICIs)^16^. Participants with low MHC I expression exhibited significantly higher CD4^+^ T cell scores, as measured by BayesPrism^17^ estimates (Fig. 5a). This was further supported by a significant negative correlation between mean MHC I expression and CD4^+^ T cell scores across these datasets (Fig. 5b).

**Extended Data Fig. 5:**
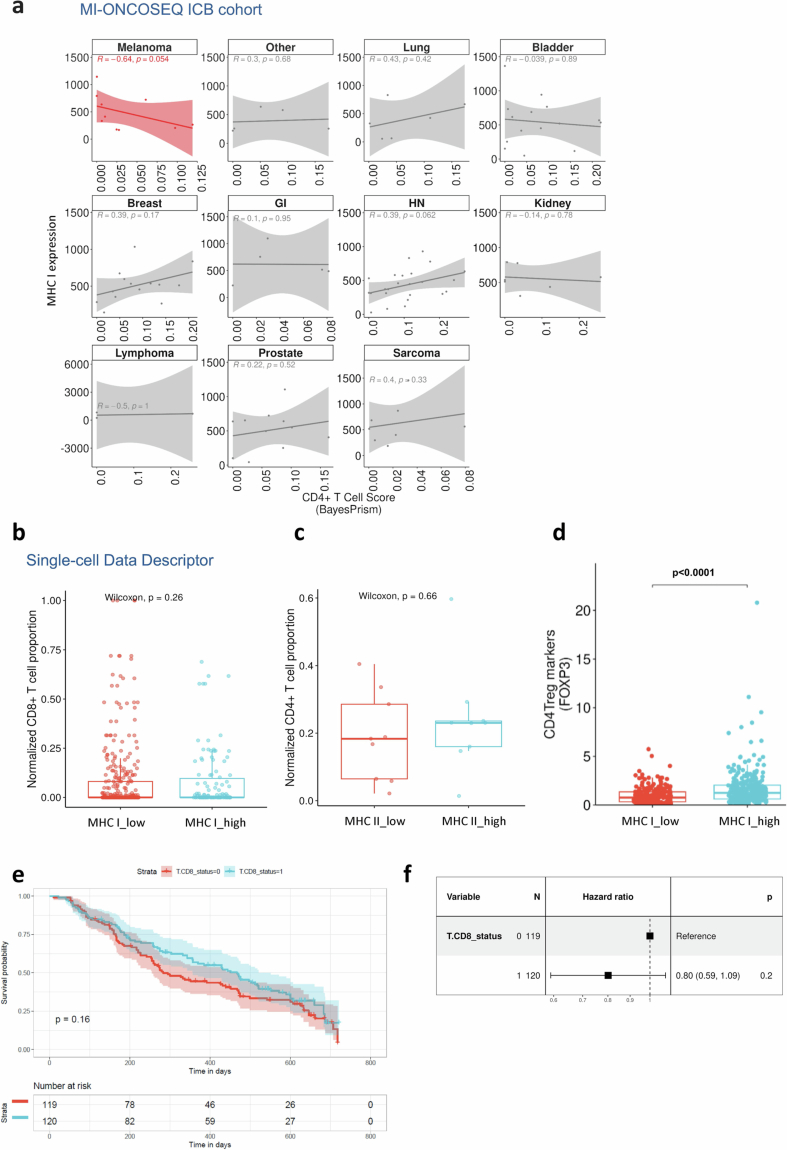
Correlation Between CD4^+^ T Cell Abundance and MHC Expression Across Cancer Types. **a**, Scatter plots showing the correlation between CD4^+^ T cell scores (estimated using BayesPrism) and MHC I gene expression levels across various cancer types in bulk RNA-seq datasets (MI-ONCOSEQ ICB cohort). Each panel represents a specific cancer type. Linear regression lines with 95% confidence intervals are shown. Spearman correlation coefficients (R) and p-values are included for each cancer type. **b**, Normalized CD8^+^ T cell proportions between MHC I-low and MHC I-high patient groups across scRNA-seq datasets from melanoma and basal cell carcinoma. (two-sided Wilcoxon test, p = 0.26). **c**, Normalized CD4^+^ T cell proportions between MHC II-low and MHC II-high patient groups in scRNA-seq datasets. Statistical significance was determined using a Wilcoxon test (p = 0.66). **d**, FOXP3 expression between MHC I-low and MHC I-high groups in the bulk ICB melanoma cohort (TIGER dataset) (two-sided Wilcoxon test, p = 4.86 × 10^⁻9^). **e**, Kaplan-Meier survival curves for patients stratified by CD4^+^ T cell abundance status, showing overall survival across time (two-sided log-rank test, p = 0.16). **f**, Forest plot summarizing a multivariable Cox regression analysis examining the association between CD8^+^ T cell status and survival outcome, reporting hazard ratio (HR), 95% confidence interval, and p-value for the variable. Box plots show the median (center line), 25th–75th percentiles (box limits), and min/max values within 1.5 × IQR (whiskers). Each point represents an independent tumor sample.

Next, to validate these observations across a broader range of cancer types, we analyzed the MI-ONCOSEQ ICB cohort^18^, which includes pretreatment tumor sequencing data from 108 participants across 11 cancer types. While melanoma showed a negative correlation, other cancer types exhibited variable trends (Extended Data Fig. 5a), underscoring the complexity of immune interactions in different cancers and the need for cancer-specific analyses.

To address cellular heterogeneity, we used our previously published data descriptor^19^ and analyzed nine datasets across melanoma^20,21^ and basal cell carcinoma^22^, confirming a significant inverse correlation between MHC I expression and normalized CD4^+^ T cell abundance (Fig. 5c,d). This finding reflects a genuine difference in immune cell composition rather than a bulk-averaging effect. Importantly, there was no significant association between MHC I expression and CD8^+^ T cell abundance or between MHC II expression and CD4^+^ T cells (Extended Data Fig. 5b,c). Although scRNA-seq resolution limited direct regulatory T cell (Treg) subsetting, analysis of bulk ICB melanoma cohort from TIGER database revealed that significantly lower FOXP3^+^CD4^+^ Tregs in the MHC I-low group compared to the MHC I-high group, indicating a lower abundance of Tregs in MHC I-low tumors (Extended Data Fig. 5d). This suggests that the increased CD4^+^ T cell infiltration observed in MHC I-low tumors is composed of CD4^+^ effector cells rather than the immunosuppressive CD4^+^ Tregs. Collectively, these results strengthen the conclusion of inverse correlation between CD4^+^ T cell infiltration and MHC I expression in persons with cancer.

Next, we next analyzed the colon cancer scRNA-seq dataset from Pelka et al.^23^. The data were analyzed separately for mismatch-repair-deficient and mismatch-repair-proficient (MMRd and MMRp) cancers because colon cancer exhibits ICI responsiveness on the basis of the MMR status. Notably, only the MMRd samples, which are immune hot and hypermutated, showed a strong association between MHC I-low expression and CD4^+^ T cell abundance but not the MMRp samples, which are relatively immune cold (Fig. 5e).

We next analyzed whether the inverse correlation between MHC I expression and CD4^+^ T cell infiltration is reflective of enhanced CD4^+^ T cell-mediated cytotoxicity of MHC I-low cancers and, therefore, clinically relevant by determining whether it correlated with improved survival. To this end, we analyzed survival data from the TIGER database. Our analysis revealed that participants with MHC I-low tumors with higher CD4^+^ T cell infiltration had significantly better survival than those with lower CD4^+^ T cell infiltration (Fig. 5f,g). Notably, the lack of a significant correlation between survival and CD8^+^ T cell levels confirmed the specificity of this finding (Extended Data Fig. 5e,f). Collectively, these human data suggest that MHC I-low tumors are more susceptible to CD4^+^ T cell-mediated immune control.

### Ferroptosis and MHC I-low tumors in human studies

**Fig. 6:**
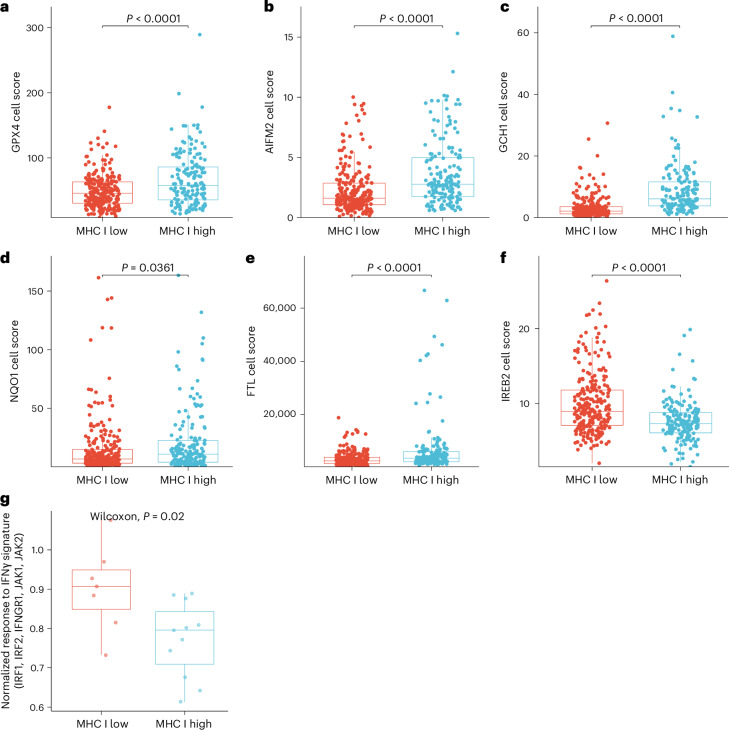
Reduced expression of anti-ferroptotic regulators and altered immune signatures in MHC I-low melanoma tumors. **a**–**f**, The expression of GPX4 (**a**), AIFM2 (**b**), GCH1 (**c**), NQO1 (**d**), FTL (**e**) and IREB2 (**f**) in MHC I-low versus MHC I-high tumors in bulk RNA-seq datasets. A two-sided unpaired *t*-test was used to test significance (GPX4, *P* = 1.56 × 10^−6^; AIFM2, *P* = 2.83 × 10^−^^8^; GCH1, *P* = 6.95 × 10^−^^15^; NQO1, *P* = 0.0361; FTL, *P* = 5.51 × 10^−5^; IREB2, *P* = 9.92 × 10^−12^), with *n* = 165 in the MHC I-high group and *n* = 249 in the MHC I-low group. **g**, Normalized interferon response signatures between MHC I-low and MHC I-high participant groups in single-cell ICB. A two-sided Wilcoxon rank-sum test was used to test significance (*P* = 0.02), with *n* = 7 in the MHC I-low group and *n* = 11 in the MHC I-high group. Box plots show the median (center line), 25th–75th percentiles (box limits) and minimum and maximum values within 1.5× the IQR (whiskers). Each point represents an independent tumor sample.

**Extended Data Fig. 6:**
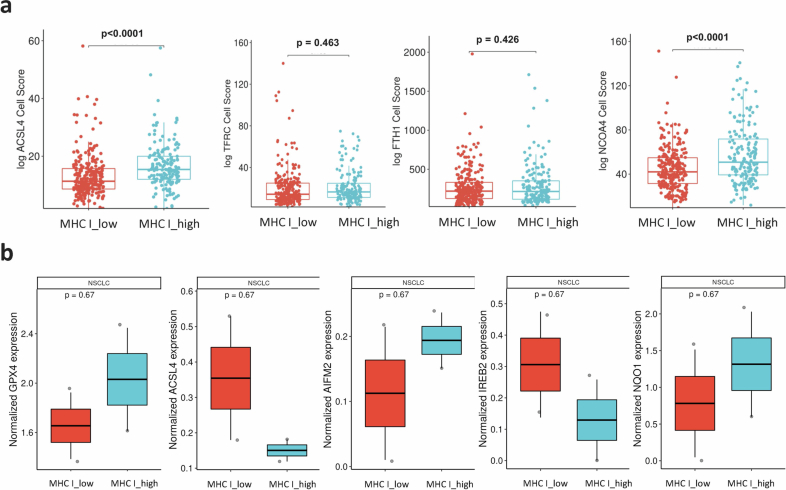
Ferroptosis and Iron Metabolism Marker Expression in Relation to MHC I Status Across Tumor Types. **a**, The expression of ACSL4 (p = 5.2×10^−6^), TFRC (p = 0.463), FTH1 (p = 0.426), and NQO1(p = 7.4×10^−7^) in MHC I-low versus MHC I-high tumors in bulk RNA-seq datasets. Two-sided unpaired t test was used. **b**, Normalized gene expression of GPX4, ACSL4, AIFM2, IREB2, NQO1 in non-small cell lung cancer (NSCLC). Box plots show the median (center line), 25th–75th percentiles (box limits), and min/max values within 1.5 × IQR (whiskers). Each point represents an independent tumor sample.

Building on evidence from animal models that MHC I-low tumors were more susceptible to CD4^+^ T cell-mediated ferroptosis, we next analyzed human datasets for ferroptosis-related molecular signatures. Our analysis revealed a molecular signature indicative of enhanced ferroptosis in MHC I-low tumors. Specifically, we showed that several MHC I-low tumors exhibited significantly lower expression of several anti-ferroptotic regulators (*GPX4*, *AIFM2*, *GCH1* and *NQO1*; Fig. 6a–d) and altered iron homeostasis markers (*FTL* and *IREB2*; Fig. 6e,f), while some other markers related to ferroptosis and iron metabolism did not show a significant or consistent correlation (Extended Data Fig. 6a). Furthermore, consistent with proposed mechanism of increased IFN response, scRNA-seq confirmed a significantly higher IFNγ response signature in MHC I-low tumor cells (Fig. 6g), demonstrating a correlation with enhanced IFNγ signaling and a proferroptotic phenotype in MHC I-low tumors.

We also extended our analysis to a pan-cancer cohort to investigate whether the proferroptotic signature in MHC I-low tumors is a universal phenomenon. While trends were variable across cancer types, we observed a proferroptotic signature in non-small cell lung cancer, a highly immune-susceptible tumor (Extended Data Fig. 6b). In this cohort, key ferroptosis regulators such as GPX4, ACSL4, AIFM2, IREB2 and NQO1 were altered in a trend consistent with a proferroptotic state in MHC I-low tumors.

Collectively, these human tumor immunotherapy datasets align with the data from the experimental models demonstrating a role for MHC I in regulating CD4^+^ T cell-mediated immunity and underscore the complex interplay between CD4^+^ T cell abundance and MHC I expression across different cancer types and molecular subtypes, emphasizing the need for subtype-specific therapeutic strategies in immunotherapy.

## Discussion

Our study provides new insights into the role of MHC I in CD4^+^ T cell-mediated immunity, unveiling mechanisms distinct from its canonical role in antigen presentation to CD8^+^ T cells. We demonstrate that the absence of surface expression of MHC I in the target cells increases susceptibility to CD4^+^ T cell-mediated cytotoxicity across alloimmunity (GVHD) and tumor immunity models and the results correlated in ICI-treated participants.

Using allogeneic HSCT models, our study reveals that the absence of β2m exclusively in host target (GI tract) cells exacerbates CD4^+^ T cell-mediated GVHD mortality in an MHC II-disparate allogeneic HSCT. This effect occurred despite the similar levels of CD4^+^ T cell activation and inflammation, demonstrating greater sensitivity of β2m-deficient target cells to cytopathic CD4^+^ T cells. Functional and mechanistic studies reveal that the enhanced sensitivity of the GVHD target intestinal cells in the absence of β2m is at least in part dependent on their greater susceptibility to iron-dependent cell death, ferroptosis. Transcriptomic analysis demonstrated similar frequency of IFNγ^+^CD4^+^ T cells but an increase in IFNγ signature in the target tissues, suggesting an enhanced response to IFNγ in the absence of β2m. Functional studies with the addition of exogenous IFNγ or in culture with MHC II-disparate allogeneic bm12 T cells showed greater lipid peroxidation in the β2m target cells, while in vivo blockade of IFNγ demonstrated a reduction in intestinal ferroptosis of MHC I-deficient allogeneic HSCT recipients.

β2m deficiency can potentially impact both classical and nonclassical MHC I function. However, the greater severity of GVHD in Tap1-KO hosts caused by MHC II-disparate donors demonstrates that enhanced sensitivity is not merely limited to β2m deficiency but is a consequence of deficiency in the MHC I. Thus, our data demonstrate an increase in allogeneic CD4^+^ T cell-mediated cell death in the intestinal epithelial target cells that lack MHC I, which is caused in part because of their increased sensitivity to IFNγ-mediated ferroptosis. Moreover, although both TAP1 and β2m deficiencies impair MHC I expression, they are mechanistically distinct. Nonclassical CD1d molecules are β2m dependent but TAP independent and certain classical MHC I molecules can present TAP-independent peptides^24^. Whether such distinct β2m-independent or TAP-independent pathways will produce a similar phenotype remains to be investigated.

Our data suggest that deficiency of MHC I enhances the sensitivity of both normal (nontransformed) IECs and the murine tumor cells to CD4^+^ T cells, suggesting that this is a broad immunological phenomenon that has implications for all CD4^+^ T cell-dependent immune responses. Importantly, enhanced sensitivity is specific to CD4^+^ T cell-mediated stress because MHC I-deficient cells did not show increased vulnerability to nonspecific inflammation, as demonstrated by DSS colitis and radiation-induced inflammation and was independent of ‘missing self’ NK activity. Crucially, the enhanced cell death remained dependent on MHC II-restricted antigen recognition as shown by both the alloimmunity and the tumor immunity models, indicating the absence of β2m does not simply reduce general cellular fitness to any stress from nonspecific inflammation but diminishes their ability to tolerate CD4^+^ T cell-mediated immunity^25^. Nonetheless, while suggestive of broad implication for all CD4^+^ T cell-mediated immune responses, these observations will need to be explored across additional tumor, autoimmunity and infection models. Additionally, future research should investigate whether the host microbiome regulates the increased CD4^+^ T cell-mediated cell death in MHC I-deficient target cells. Furthermore, while we identified ferroptosis as a key mechanism, further clinical studies still have to determine whether other metabolic or cell death pathways also contribute to the increased vulnerability of the β2m-deficient cells from CD4^+^ T cell-mediated immunity.

MHC I downregulation is among the most common escape mechanisms of tumor immunotherapy. However, some participants, despite the downregulation of MHC I on tumor cells, respond to ICI therapy. Our data combined bulk and single-cell transcriptomic analyses from participants may help explain the observed efficacy of ICI therapy in this subset of participants. Specifically, CD4^+^ T cell infiltration significantly increased in participants with melanoma exhibiting low MHC I expression but not in participants with normal MHC I expression treated with ICI therapy. While the proportion of Tregs was not enhanced, the survival of participants who received ICI therapy was associated with reduced MHC I expression, which correlated with greater CD4^+^ T cell infiltration. Notably, these participants responded and did not show a change in CD8^+^ T cell infiltration. Similar significant correlations were also noted in participants with MMRd colon cancer between low MHC I and CD4^+^ T cell infiltration and responses, while a statistically insignificant trend was observed in other cancers where the participant numbers were small. These data will need to be validated in larger datasets and in prospective trials in the future. Nonetheless, our observations demonstrate the generalizability and validation of the experimental findings and challenge the existing paradigm by extending the functional relevance to MHC I for CD4^+^ T cell-mediated tumor immunity beyond its role of antigen presentation to CD8^+^ T cells. Our data suggest that developing and designing immunotherapeutic strategies to enhance or leverage CD4^+^ T cells could be used to proactively tailor the treatment of MHC I-downregulated tumors, which are often considered ‘immune-cold’ tumors.

Our data provide insights that have additional potential clinical implications. While pathogens and tumor cells often downregulate MHC I-mediated antigen presentation to escape from immune surveillance, our observations now suggest that this deficiency may paradoxically sensitizes them to CD4^+^ T cell-mediated elimination. This implication is borne out by our demonstration of lower relapse rates and more significant CD4^+^ T cell infiltration after ICI therapy despite the downregulation of MHC I in tumors. This is also suggested, albeit indirectly, by the recent report by C. June’s group, which showed the emergence and persistence of a highly activated cytotoxic CD4^+^ population from adoptively transferred CD19-redirected chimeric antigen receptor T cells in persons with long-term remission^26^. Our data also provide a potential explanation for the role of MHC II, human leukocyte antigen (HLA)-DP mismatch and MHC II expression on leukemic cells in regulating relapse rates after clinical allogeneic HSCT^27–29^. This will require confirmation by future studies with engineered or mismatched antigen-specific CD4^+^ T cells against immune-cold solid and hematological cancers with downregulation or loss of MHC I. Similarly, given the role of MHC I downregulation by viruses and microbes, our data may also have direct implications for anti-infectious immunity, which future studies must directly confirm.

In GVHD, beyond apoptosis, we now identify ferroptosis as a contributor to the severity of GI-GVHD. Whether ferroptosis contributes to other target organ damage must be determined in future studies. Nonetheless, our observations provide biological insights into the clinical correlation between iron overload and GVHD and a recent report demonstrating a potential GVHD-reducing impact of iron chelation^30^. Our data also extend the recent observations of the contribution of ferroptosis to CD4^+^ T cell-mediated immunotherapy^31^ and indicate that it will be especially relevant in the context of the downregulation of MHC I. The mechanism by which CD4^+^ T cells cause greater ferroptosis in the MHC I-deficient targets is at least partly dependent on IFNγ. Future studies must determine the mechanisms of enhanced ferroptosis induction by CD4^+^ T cells in the absence of MHC I.

Beyond its well-studied role in infectious, tumor and alloimmunity, MHC I is also known to have a role in disease susceptibilities such as ankylosing spondylitis, primary hemochromatosis and other inherited conditions. It is important to note that our data should not be construed to indicate that CD4^+^ T cells can compensate for the absence of CD8^+^ T cells. This is directly demonstrated in participants with type 1 bare lymphocyte syndrome^32,33^, an autosomal recessive disease characterized by a deficiency of MHC I and characterized by recurrent microbial infections. Instead, it suggests that these participants may be more resistant to a broad range of infectious agents whose antigens are presented to CD4^+^ T cells in an MHC II-restricted manner. Furthermore, it is also possible that diseases susceptible to MHC I could be regulated by leveraging CD4^+^ T cells. Nonetheless, our data complement and extend the literature demonstrating noncanonical roles of MHC I beyond its canonical roles in antigen presentation to CD8^+^ T cells or in missing self recognition by NK cells, in the regulation of CD4^+^ T cell-mediated immunity. Thus, we expand the scope of MHC I from the long-held paradigm in T cell immunity that MHC I exclusively mediates only CD8^+^ T cell responses.

## Methods

### Mice

C57BL/6 mice were purchased from Charles River. B6.Cg-*Rag1*^*tm1Mom*^*Tyrp1*^*B−w*^Tg(Tcra,Tcrb)9Rest/J (008684), B6(C)-*H2-Ab1*^*bm12*^/KhEgJ (001162), B6.129P2-*B2m*^*tm1Unc*^/DcrJ (002087), B6(Cg)-*B2m*^*tm1c(EUCOMM)Hmgu*^/J (034858), B6.Cg-Tg(Vil1-cre)997Gum/J (004586), B6;129S2-*Tap1*^*tm1Arp*^/J (002458), STOCK*Gpx4*^*tm1.1Qra*^/J (027964) and B6.Cg-Tg(Vil1-cre/ERT2)23Syr/J (020282) mice were purchased from The Jackson Laboratory. β2m floxed mice were bred with Vil-Cre mice to generate β2m^fl/fl^Vil-Cre^+^ mice. β2m^fl/^^fl^Vil-Cre^+^ mice are referred to as β2m^∆IEC^ mice. *Gpx4*^*fl/fl*^ mice were bred with Vil1-cre/ERT2 mice to generate a conditional KO model. Mice receiving tamoxifen injections were designated as Gpx4^∆IEC^, while corn-oil-treated Gpx4^*fl/fl*^ littermates served as WT controls. The age of mice used for experiments ranged between 7 and 12 weeks. Mice were housed in a specific-pathogen-free facility under a 12-h light–dark cycle. Ambient temperature was maintained at 20–24 °C with a relative humidity of 45–65%. Mice had ad libitum access to standard chow and water throughout the study. All animals were cared for under regulations reviewed and approved by the University Committee on Use and Care of Animals of the University of Michigan and Baylor College of Medicine.

### HSCT

HSCTs were performed as previously described^34^. Briefly, we used an MHC-matched, syngeneic (C57BL/6 → C57BL/6) model and an MHC II-mismatched, allogeneic (bm12 → C57BL/6) model^14,35–41^. On day −1, mice on a C57BL/6 background received a total of 1,000 cGy of irradiation (single or split dose separated by 4 h). Donor splenic T cells were enriched using the pan T cell isolation kit II and manual magnetic-activated cell sorting (MACS) with LS columns (Miltenyi Biotec). A total of 2 × 10^6^–2.5 × 10^6^ T cells and 5 × 10^6^ whole BM cells were transferred to recipients on day 0. For the NK cell experiment, NK cells were depleted from the graft through magnetic labeling of NKp46 microbeads (130-095-390, Miltenyi Biotec) and NK cells were depleted from recipients through intraperitoneal injection of 200 μg of anti-NK1.1 antibody (PK-136, BioXcell) 1 day before cell transfer. Survival was monitored daily and the recipient body weight and GVHD clinical scores were determined weekly^34,42^. Animals received vehicle or DFX (20 mg kg^−1^; SML2673-50, Sigma-Aldrich) through a flexible 20G 1.5-inch intragastric gavage needle daily for 5 days before BM transplant (BMT) and 10 days after HSCT. Mice were injected with the 200 μg of anti-IFNγ antibody intraperitoneally on days 0, 2, 4 and 6 after HSCT.

### Cells and CRISPR KO

**Extended Data Fig. 7:**
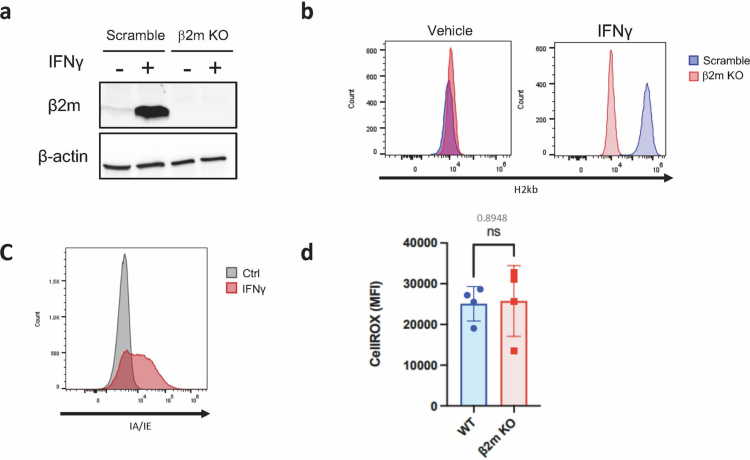
Validation of experimental models and knockout efficacy. **a**, β2m expression was measured by Western blotting in scramble or β2m KO B16 cell lines with or without IFNγ treatment overnight. **b**, H2kb surface expression was measured by flow cytometry on scramble or β2m KO B16 cell lines with or without IFNγ treatment overnight. **c**, MHC II (IA/IE) surface expression was measured by flow cytometry on B16 cell lines with or without IFNγ treatment overnight. **d**, Oxidative stress was measured with CellRox in IECs isolated from allogeneic WT or β2m KO recipients seven days after HSCT. Two-tailed unpaired t-test (**d**) was used to determine significance. Source data

B16-F0 cells (B16, CRL-6322) were purchased from the American Type Culture Collection. We confirmed that MHC II expression can be induced in B16 cells by IFNγ treatment (Extended Data Fig. 7c), consistent with previous findings^11,43^. To generate scramble or β2m-KO cell lines, B16 were nucleofected on the nucleofector X machine with plasmid containing two guide RNAs (gRNAs), Cas9, puromycin resistance gene and eGFP. For β2m KO, gRNAs were AGTATACTCACGCCACCCAC and CCGAGCCCAAGACCGTCTAC. A scramble gRNA control vector (pRP[CRISPR]-EGFP/Puro-hCas9-U6>Scramble_gRNA1; VectorBuilder, ID: VB010000-9354ztt) was used as a control.

### Tumor

Mice were injected s.c. with 2.5 × 10^5^ B16 tumor cells on day −10. On day 0, mice were treated with 500 cGy of total body irradiation (TBI). Then, 4 h later, mice were injected intravenously (i.v.) with 50,000 CD4^+^Trp1^+^ cells. CD4^+^Trp1^+^ for adoptive transfers was isolated from Trp1 mice using CD4 magnetic beads (Miltenyi Biotec).

### IEC isolation

Primary IECs were obtained from C57BL/6 mice as described previously^1^. Briefly, luminal contents of intestine were flushed with CMF buffer (Ca^2+^/Mg^2+^-free Hanks’ balanced salt solution (14185052, Thermo Fisher Scientific) supplemented with 25 mM sodium bicarbonate (S6014, Sigma-Aldrich) and 2% FBS (100-106, Gemini Bio Products)). The intestine was cut longitudinally, then minced into 50-mm pieces, washed with CMF six times, transferred to CMF with 5 mM EDTA (51201, Lonza) and incubated at 37 °C for 45 min (shaking tubes every 15 min). Supernatant containing IECs was then transferred through a 100-μm cell filter followed by incubation on ice for 10 min to allow sedimentation. Supernatant was again transferred through a 70-μm cell filter.

### Intestinal lamina propria leukocyte isolation

The small intestine was isolated, cut longitudinally and washed twice in PBS. The tissue was then chopped into 50-mm pieces and epithelial and intraepithelial cells were separated from the underlying lamina propria by incubation in CMF buffer containing 30 mM EDTA and 1 mM DTT at 37 °C for 10 min with shaking. After pulse-vortexing and washing in PBS twice, the tissue was transferred to RPMI with 10% FBS, 100 U per ml collagenase type VIII and 150 µg ml^−1^ DNase I at 37 °C for 1 h with shaking. Leukocytes were isolated from the supernatant using a Percoll gradient separation method, in which the cells were resuspended in 40% Percoll and underlayered with 80% Percoll, followed by centrifugation at 860*g* for 20 min without a brake. The interface was then collected for fluorescence-activated cell sorting.

### BMDC culture

Whole BM was isolated from femurs and tibias of C57BL/6 or β2m-KO mice, cultured with murine recombinant granulocyte-macrophage colony-stimulating factor (GM-CSF; 20 ng ml^−1^; PeproTech) for 7 days and then isolated.

### BMDC culture and isolation

To obtain BMDCs, BM cells from WT B6 or β2m-KO mice were cultured with murine recombinant GM-CSF (20 ng/ml; PeproTech) for 7 days and harvested. BMDCs were isolated using CD11c microbeads and LS MACS columns (Miltenyi Biotec). Isolated BMDCs were then stimulated with lipopolysaccharide (LPS; 0.5–5 ng ml^−1^; Invivogen) for 16 h. Supernatant was collected for cytokine measurement and BMDCs were harvested for flow cytometry.

### Mixed lymphocyte reaction (MLR)

Splenic T cells from bm12 mice animals were used as responders and BMDCs from of C57BL/6 or β2m-KO mice were used as stimulators in an MLR. To create different responder-to-stimulator ratios, 2 × 10^4^, 1 × 10^4^ or 5 × 10^3^ BMDCs were cocultured with 2 × 10^5^ bm12 T cells in a 96-well round-bottom plate for 72 and 96 h. The incorporation of ^3^H-thymidine (1 μCi per well) by proliferating T cells during the final 16 h of coculture was measured using a Betaplate reader (Wallad).

### Cytokine ELISA

Supernatants from cell culture were harvested and analyzed for TNF (BD Biosciences) and IL-6 (BD Biosciences) according to the manufacturer’s instructions. Absorbance measurements was performed on a SpectraMax iD5 multimode microplate reader using SoftMax Pro version 7.1 software (Molecular Devices).

### CTL assay

Splenic T cells from bm12 mice and LPS-stimulated BMDCs from WT B6 mice were cocultured in a 10:1 ratio for 7 days. T cells were purified with a CD90.2 T cell isolation kit (Miltenyi Biotec) and used as effector cells. A total of 2 × 10^6^ B16 cells were incubated with 2 μBq of Na_2_^51^CrO_4_ (NEZ030001MC, PerkinElmer) for 2 h at 37 °C in a 5% CO_2_ atmosphere and were used as target cells. After washing, 4,000 labeled target cells were resuspended, added to triplicate wells at varying E:T ratios and then incubated for 6 h. Maximal or minimum release was determined by the addition of Triton X-100 or medium alone to targets, respectively. After incubation, supernatants were transferred to a Lumaplate (600633, PerkinElmer) and ^51^Cr activity was determined using Top Count NXT (Hewlett Packard).

### Flow cytometry

Cells were resuspended in 2% BSA in PBS and stained with the following antibodies and reagents: anti-CD80–FITC (16-10A1, 1:200; Biolegend, 104706), anti-CD40–PE (FGK45, 1:500; Biolegend, 157506), anti-NK1.1–PE (PK-136, 1:100; Biolegend, 108708), anti-CD4–violetFluor 450 (GK1.5, 1:200; Tonbo, 75-0041), anti-CD8–violetFluor 500 (2.43, 1:200; Tonbo, 85-1886), anti-CD27–Pacific blue (LG.3A10, 1:500; Biolegend, 124218), anti-CD44–PerCP–Cy5.5 (IM7, 1:200; Biolegend, 103032), anti-CD45–PE (30-F11, 1:200; Biolegend, 103106), anti-CD62L–PE (MEL-14, 1:200; Biolegend, 104408), anti-CD69–violetFluor 450 (H1.2F3, 1:100; Cytek Biosciences, 75-0691), anti-Foxp3–PerCP–eFluor710 (FJK-16s, 1:200; eBioscience, 46-5773-82), anti-GzmB–FITC (GB11, 1:100; Biolegend, 515403), anti-Klrg1–FITC (2F1, 1:500; Biolegend, 138410), anti-Ki67–Pacific blue (16A8, 1:100; Biolegend, 652422), anti-Rorgt–PE (AFKJS-9, 1:200; eBioscience, 12-6988-82), anti-Tbet–PE–Cy7 (4B10, 1:200; Biolegend, 644823), anti-IFNg–APC (XMG1.2, 1:100; Biolegend, 505810), anti-TNF–BV711 (MP6-XT22, 1:100; Biolegend, 506349), anti-IL-1β–eFluor450 (NJTEN3, 1:100; eBioscience, 48-7114-82), anti-IL-10–PE (JES5-16E3, 1:100; Biolegend, 505008), anti-CD326–FITC (G8.8, 1:200; Biolegend, 118208), anti-CD326–BV711 (G8.8, 1:200; Biolegend, 118233), anti-annexin V–APC (1:20; Biolegend, 640920), anti-H2kb–BV421 (AF6-88.5, 1:100; Biolegend, 116525), anti-IA/IE–APC (M5/114.15.2, 1:100; Biolegend, 107614). For surface immunophenotyping, single-cell suspensions were incubated with the relevant antibodies for 30 min at 4 °C, washed and fixed with fixation buffer (Invitrogen, 00-8222-49) before analysis. Intracellular and nuclear staining was performed following surface staining and fixation in permeabilization buffer (Thermo Fisher, 00-5521-00) for 30 min at room temperature. For lipid peroxidation measurement, cells were incubated with 1 μM C11 BODIPY 581/591 for 30 min at 37 °C. Cells were washed and then run on a flow cytometer. Lipid peroxidation was determined by dividing the median fluorescence intensity (MFI) of the FITC channel by the MFI of the PE channel. Cells were acquired on an Attune NxT flow cytometer or a Cytek Northern Lights system using SpectroFlo version 3.3.0 software (Cytek Biosciences) and analyzed using FlowJo (version 10.10.0).

### Immunoblot analysis

B16 cell lines or IECs isolated from GVHD recipients were lysed in RIPA buffer (Sigma-Aldrich, R0278). Equal amounts of proteins were loaded on an SDS–PAGE gel (Bio-Rad, 4568124), electrophoresed and transferred to a PVDF membrane (Bio-Rad, 1704151). Blots were incubated with anti-ACSL4 (Abcam, ab155282; 1:3,000), IRF1 (Cell Signaling Technology, 8478T; 1:1,000), and β-actin–horseradish peroxidase (HRP) (Cell Signaling Technology, 5152S; 1:1,000) primary antibodies overnight at 4 °C. On the next day, blots were washed and incubated with secondary anti-rabbit–HRP (GenDEPOT, SA002-500) at room temperature for 1 h. Bound antibody was detected with Clarity Max western enhanced chemiluminescence substrate (100 ml; Bio-Rad, 1705062) and captured using a ChemiDoc MP Imaging system with Image Lab version 3.0.1.14 software (Bio-Rad).

### RNA isolation and qPCR

Total RNA from single-cell suspensions was isolated using the RNeasy Kit (Qiagen, 74104). Reverse transcription and qPCR was performed using an iTaq Universal SYBR green one-step kit (Bio-Rad, 1725151) according to the manufacturer’s instructions. qPCR was performed using a CFX Opus 96 real-time PCR system and data were collected with CFX Maestro version 2.3 software (Bio-Rad). The following primers were used to detect the following transcripts: 5′- ATTGGTCAGGGATATGGGCT-3′ and 5′-AGAGGAGCTCCAACTCTTCCA-3′ (*Acsl4*); 5′- TCCAAGTCCAGCCGAGACACTA-3′ and 5′-ACTGCTGTGGTCATCAGGTAGG-3′ (*Irf1*); 5′- GAGCTGTTTGCAGACAAAGTTC-3′ and 5′-CCCTGGCACATGAATCCTGG-3′ *(Ppia*).

### Malondialdehyde (MDA) assay

Tissue samples were digested using the OMNI bead disruptor. A lipid peroxidation MDA assay kit (Abcam, ab118970) was used according to the manufacturer’s instructions.

### Iron measurements

Cheek bleeds were used to obtain blood for serum iron measurements. Serum iron was quantified using the iron assay kit (Abcam, ab83336) according to the manufacturer’s instructions. For tissue iron measurements, mice were killed and perfused by transcardiac perfusion with PBS without Ca^2+^ and Mg^2+^. The heart, liver and small intestine were isolated and lyophilized. Precisely weighed samples were completely digested with H^16^NO_3_ and then redissolved into 10 ml of 2% HNO_3_ after drying down the concentrated HNO_3_. The dissolved sample solutions were analyzed for iron with an Agilent 725 inductively coupled plasma (ICP) optical emission spectroscope in the ICP Research Lab at the University of Houston. Iron concentrations were calibrated with a series of iron solution standards (0.1, 0.5, 5 and 50 ppm).

### DSS colitis

For the DSS colitis model, animals were provided with drinking water containing 3% DSS (AAJ6360622, Fisher Scientific) for 7 days. Body weight and condition were observed and recorded daily. Mice were taken off DSS water for 3 days and then killed for endpoint analyses.

### FITC–dextran assay

Food and water was withheld from all mice for 4 h on day 21 after BMT. FITC–dextran (Sigma-Aldrich) was administered through a 20G 1.5-inch flexible intragastric gavage needle (Braintree Scientific) at a concentration of 50 mg ml^−1^ in PBS. BMT recipients received 800 mg kg^−1^ (~16 mg per mouse). Then, 4 h later, serum was collected from peripheral blood and diluted 1:1 with PBS; fluorescence measurements was performed on a SpectraMax iD5 multimode microplate reader using SoftMax Pro version 7.1 software (Molecular Devices) at excitation and emission wavelengths of 485 and 535 nm, respectively. Concentrations of FITC–dextran experimental samples were determined using a standard curve.

### scRNA-seq

Libraries were prepared from a single-cell suspension of IECs using 10x Chromium Next-GEM single-cell 3’ GEM version 3.1 library prep. Libraries were sequenced on a NovaSeq X Plus 25B for 2× 150-bp reads. On day 6 after transplant, four biological replicates each of Cre^−^ and Cre^+^ mice were collected for analysis. Data processing and downstream analyses were performed in R version 4.4.2 using Seurat version 5.3.1, DESeq2 version 1.46.0, clusterProfiler version 4.14.6, org.Mm.eg.db version 3.20.0, AnnotationDbi version 1.68.0 and ggplot2 version 4.0.1. Three gene sets were curated for functional scoring in IECs: (1) IFNγ-related antigen presentation (*H2-K1*, *H2-D1*, *B2m*, *Tap1*, *Tap2*, *Psmb8*, *Psmb9*, *Cd74* and *Ciita*)^44^; (2) IFNγ-related chemokines and signaling (*Cxcl9*, *Cxcl10*, *Cxcl11*, *Stat1*, *Gbp2*, *Irf1* and *Socs1*)^45^; and (3) ferroptosis activation (*Irf1*, *Stat1*, *Jak1*, *Jak2*, *Acsl4*, *Alox12*, *Alox15*, *Lpcat3*, *Sat1*, *Ptgs2*, *Nox1*, *Tfrc*, *Ncoa4*, *Fth1* and *Ftl1*)^15,46^. In addition, activation marker gene sets for immune cells were curated: (1) CD4 T cells (*Stat1*, *Mal*, *Socs1*, *Il2*, *Odc1*, *Psat1*, *Pycr1*, *Tnf*, *Mir155hg*, *Nme1*, *Maf* and *Tnfrsf18*)^47^; (2) CD8 T cells (*Gzmb*, *Ifng*, *Ccl4*, *Ccl3*, *Xcl1*, *Ifit3*, *Hopx*, *Rcan2* and *Prf1*)^48^; (3) NK cells (*Prf1*, *Gzmb*, *Gzma* and *Nkg7*)^48^; (4) Macrophages (*Nfkb1*, *Junb*, *Creb1*, *Stat3*, *Mmp9*, *Csf1*, *Fabp5* and *Tnfaip6*)^49^; and (5) neutrophils (*Gbp5*, *Fcgr1*, *Mpo*, *Mmp9*, *Padi4*, *Itgam* and *Fcgr3*)^50^. Module scores for each curated gene set were calculated in Seurat using the AddModuleScore function and were applied for subsequent cell-type-specific comparisons.

### Bulk ICB dataset analysis

To assess the role of CD4^+^ T cells in bulk ICB cohorts, we retrieved five melanoma RNA-seq datasets from the TIGER database^16^, specifically GSE115821 (ref. ^51^), GSE78220 (ref. ^52^), GSE91061 (ref. ^53^), phs000452 (ref. ^54^) and PRJEB23709 (ref. ^55^). R version 4.1.3 was used in this analysis. For the evaluation of the immune compartment, we used BayesPrism^17^ (version 2.2.2) with an scRNA-seq melanoma study by Jerby-Arnon et al.^21^ as a reference. The Jerby-Arnon et al. datasets for melanoma samples were obtained from the Gene Expression Omnibus (GEO) under accession number GSE115978. This study builds upon the previously published melanoma study by Tirosh et al.^20^, thereby incorporating the previous samples. Metadata were also sourced from the Tumor Immune Single-Cell Hub^56^. The raw count data and cell annotation files were converted into Seurat objects^57^ (version 4.1.0). An arbitrary threshold was applied to categorize participants into MHC I-low and MHC I-high groups on the basis of the mean expression of MHC I genes, calculated as the average expression of HLA-A, HLA-B and HLA-C. We visualized the results using the ggplot2 package (version 3.3.6).

For additional validation, we analyzed a cohort treated with ICB at the University of Michigan (MI-ONCOSEQ ICB cohort), comprising 108 participants across 11 cancer types^18,58^. This dataset included pretreatment tumor sequencing data. BayesPrism was used to deconvolute these datasets, allowing us to evaluate the correlation between MHC I gene expression and estimated CD4^+^ T cell abundance.

### Single-cell ICB dataset analysis

To explore this relationship in ICB single-cell data, we leveraged our previously published data descriptor^19^, which encompasses nine cancer types. We subsetted the datasets to include only those with CD4^+^ T cell annotations, resulting in three datasets (two melanoma datasets^20,21^ and one basal cell carcinoma dataset^22^). The normalized CD4^+^ T cell abundance was determined by calculating the ratio of total CD4^+^ T cells to total cells in each participant. For each dataset, a separate threshold was established to classify participants into MHC I-low or MHC I-high groups. Additionally, we evaluated CD8^+^ T cell estimates in relation to MHC I-low and MHC I-high classifications for control purposes. We also investigated CD4^+^ T cells in relation to MHC II-low and MHC II-high classifications.

To further investigate the single-cell expression of CD4^+^ T cells in relation to MHC I levels, we used a colon cancer scRNA-seq dataset from Pelka et al.^23^, available from the GEO under accession number GSE178341. The data were analyzed separately for MMRd, MMRp and normal samples. The ‘AverageExpression’ function was used to evaluate MHC I gene expression in each participant, generating pseudobulk expression profiles. The normalized CD4^+^ T cell abundance was calculated as the ratio of total CD4^+^ T cells to total epithelial cells in each participant.

### Statistical analysis

Data are presented as the means ± s.d. Statistical analysis and visualization were performed using GraphPad Prism (version 10.6.1). All sample sizes and statistical tests used are detailed in each figure legend. Data normality was confirmed using the Shapiro–Wilk test. Exact *P* values are provided for all comparisons where *P* > 0.0001. For values below this threshold, *P* < 0.0001 is reported.

### Reporting summary

Further information on research design is available in the Nature Portfolio Reporting Summary linked to this article.

## Online content

Any methods, additional references, Nature Portfolio reporting summaries, source data, extended data, supplementary information, acknowledgements, peer review information; details of author contributions and competing interests; and statements of data and code availability are available at 10.1038/s41590-026-02480-z.

## Supplementary information

Supplementary Information Gating strategy of CD326^+^ IECs.

Reporting Summary

## Source data

Source Data Extended Data Figs. 4 and 7 Unprocessed western blots.

## Data Availability

Mouse transcriptomics data generated in this study were deposited to the GEO database under accession number GSE316959. All human analyses used publicly available bulk RNA-seq and scRNA-seq datasets from established repositories through the GEO, Database of Genotypes and Phenotypes, European Nucleotide Archive and Zenodo under accession codes GSE115821, GSE78220, GSE91061, phs000452, PRJEB23709, GSE115978, GSE178341, phs000673.v5.p1 and 10.5281/zenodo.10407126 (ref. ^59^). Source data are provided with this paper.
