# Supplementary material for: MHC class I on target cells regulates CD4+ T cell-mediated immunity
Source: Nat Immunol. 2026 Mar 24;27(5):1000–12. doi: 10.1038/s41590-026-02480-z (PMC13132726; doi:10.1038/s41590-026-02480-z)

nature immunology

Supplementary information https://doi.org/10.1038/s41590-026-02480-z

MHC class I on target cells regulates CD4^{+} T cell-mediated immunity

In the format provided by the authors and unedited

Gating strategy of CD326+ IECs

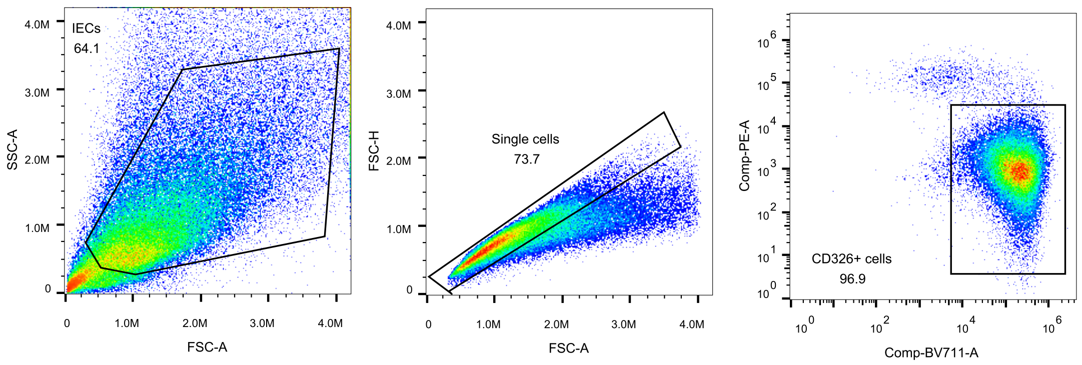

Supplement: Supplementary file 1 — Gating strategy of CD326+ IECs. [file 41590_2026_2480_MOESM1_ESM.pdf]
