# Supplementary material for: MHC class I on target cells regulates CD4+ T cell-mediated immunity
Source: Nat Immunol. 2026 Mar 24;27(5):1000–12. doi: 10.1038/s41590-026-02480-z (PMC13132726; doi:10.1038/s41590-026-02480-z)
Supplement: Supplementary file 2 — Reporting Summary [file 41590_2026_2480_MOESM2_ESM.pdf]

nature portfolio

Corresponding author(s): Pavan Reddy

Last updated by author(s): Feb 8, 2026

Reporting Summary

Nature Portfolio wishes to improve the reproducibility of the work that we publish. This form provides structure for consistency and transparency in reporting. For further information on Nature Portfolio policies, see our Editorial Policies and the Editorial Policy Checklist.

Statistics

For all statistical analyses, confirm that the following items are present in the figure legend, table legend, main text, or Methods section.

n/a	Confirmed	
[ ]	[x]	The exact sample size (*n*) for each experimental group/condition, given as a discrete number and unit of measurement
[ ]	[x]	A statement on whether measurements were taken from distinct samples or whether the same sample was measured repeatedly
[ ]	[x]	The statistical test(s) used AND whether they are one- or two-sided<br>*Only common tests should be described solely by name; describe more complex techniques in the Methods section.*
[x]	[ ]	A description of all covariates tested
[ ]	[x]	A description of any assumptions or corrections, such as tests of normality and adjustment for multiple comparisons
[ ]	[x]	A full description of the statistical parameters including central tendency (e.g. means) or other basic estimates (e.g. regression coefficient) AND variation (e.g. standard deviation) or associated estimates of uncertainty (e.g. confidence intervals)
[ ]	[x]	For null hypothesis testing, the test statistic (e.g. *F*, *t*, *r*) with confidence intervals, effect sizes, degrees of freedom and *P* value noted<br>*Give P values as exact values whenever suitable.*
[x]	[ ]	For Bayesian analysis, information on the choice of priors and Markov chain Monte Carlo settings
[x]	[ ]	For hierarchical and complex designs, identification of the appropriate level for tests and full reporting of outcomes
[x]	[ ]	Estimates of effect sizes (e.g. Cohen's *d*, Pearson's *r*), indicating how they were calculated

Our web collection on statistics for biologists contains articles on many of the points above.

Software and code

Policy information about availability of computer code

Data collection	Flow Cytometry: SpectroFlo v3.3.0 (Cytek Biosciences).<br>Western Blot: Image Lab v3.0.1.14 (Bio-Rad).<br>qPCR: CFX Maestro v2.3 (Bio-Rad).<br>Microplate Reader: SoftMax Pro v7.1 (Molecular Devices)
Data analysis	For FACS data analysis, FlowJo v10.10.0 was used. Statistical analysis was performed using GraphPad Prism v10.6.1 (GraphPad Software) to generate figures and perform statistical tests. For human transcriptomic analysis, R v4.1.3 and the following packages were used: BayesPrism v2.2.2, Seurat v4.1.0, and ggplot2 v3.3.6. For mouse transcriptomic analysis, Seurat v5.3.1, DESeq2 v1.46.0, clusterProfiler v4.14.6, org.Mm.eg.db v3.20.0, AnnotationDbi v1.68.0, and ggplot2 v4.0.1 were used. For immunoblot analysis, ImageJ v1.54g was used.

For manuscripts utilizing custom algorithms or software that are central to the research but not yet described in published literature, software must be made available to editors and reviewers. We strongly encourage code deposition in a community repository (e.g. GitHub). See the Nature Portfolio guidelines for submitting code & software for further information.

Data

Policy information about availability of data

All manuscripts must include a data availability statement. This statement should provide the following information, where applicable:

- Accession codes, unique identifiers, or web links for publicly available datasets
- A description of any restrictions on data availability
- For clinical datasets or third party data, please ensure that the statement adheres to our policy

Source data are provided with this paper. Mouse transcriptomics data generated in this study have been deposited in the NCBI Gene Expression Omnibus (GEO) database under the accession code GSE316959. All human analyses utilized publicly available bulk RNA-seq and single-cell RNA-seq datasets from established repositories via GEO, dbGaP, ENA, and Zenodo under accession codes GSE115821, GSE78220, GSE91061, phs000452, PRJEB23709, GSE115978, GSE178341, phs000673.v5.p1, and 10.5281/zenodo.10407126.

Research involving human participants, their data, or biological material

Policy information about studies with human participants or human data. See also policy information about sex, gender (identity/presentation), and sexual orientation and race, ethnicity and racism.

Reporting on sex and gender	*Use the terms sex (biological attribute) and gender (shaped by social and cultural circumstances) carefully in order to avoid confusing both terms. Indicate if findings apply to only one sex or gender; describe whether sex and gender were considered in study design; whether sex and/or gender was determined based on self-reporting or assigned and methods used.<br>Provide in the source data disaggregated sex and gender data, where this information has been collected, and if consent has been obtained for sharing of individual-level data; provide overall numbers in this Reporting Summary. Please state if this information has not been collected.<br>Report sex- and gender-based analyses where performed, justify reasons for lack of sex- and gender-based analysis.*
Reporting on race, ethnicity, or other socially relevant groupings	*Please specify the socially constructed or socially relevant categorization variable(s) used in your manuscript and explain why they were used. Please note that such variables should not be used as proxies for other socially constructed/relevant variables (for example, race or ethnicity should not be used as a proxy for socioeconomic status).<br>Provide clear definitions of the relevant terms used, how they were provided (by the participants/respondents, the researchers, or third parties), and the method(s) used to classify people into the different categories (e.g. self-report, census or administrative data, social media data, etc.)<br>Please provide details about how you controlled for confounding variables in your analyses.*
Population characteristics	*Describe the covariate-relevant population characteristics of the human research participants (e.g. age, genotypic information, past and current diagnosis and treatment categories). If you filled out the behavioural & social sciences study design questions and have nothing to add here, write "See above."*
Recruitment	*Describe how participants were recruited. Outline any potential self-selection bias or other biases that may be present and how these are likely to impact results.*
Ethics oversight	*Identify the organization(s) that approved the study protocol.*

Note that full information on the approval of the study protocol must also be provided in the manuscript.

Field-specific reporting

Please select the one below that is the best fit for your research. If you are not sure, read the appropriate sections before making your selection.

☐ Life sciences ☐ Behavioural & social sciences ☐ Ecological, evolutionary & environmental sciences

For a reference copy of the document with all sections, see nature.com/documents/nr-reporting-summary-flat.pdf

Life sciences study design

All studies must disclose on these points even when the disclosure is negative.

Sample size	The sample size of 3-6 per group were used for in vitro studies and 3-8 were used for individual in vivo studies. Sample sizes for in vitro experiments were chosen based on previous publications using similar experimental models. Sample sizes for in vivo experiments were based on extensive historical data and previous experience with these specific models in our laboratory (PMID: 33731431; 34686860; 30201970; 33463537; 33277367) to ensure adequate resolution and statistical significance between syngeneic and allogeneic control populations.
Data exclusions	No data were excluded
Replication	All in vitro experiments were independently repeated at least three times. In vivo experiments were performed using at least two independent cohorts of mice. All experimental findings were reproducible.
Randomization	For comparisons between WT and KO mice, mice were allocated based on their genotype; however, animals were age- and sex-matched, and littermates were used whenever possible to minimize biological variation. Mice from multiple litters were used to minimize litter-specific

effects, and animals were processed in a random order during data collection.
For B16-F0 tumor-bearing mice, animals were randomly assigned to different groups.
For in vitro experiments, cultured cells were randomly allocated to different treatment plates or wells from a single homogenous cell suspension to ensure consistency.

Blinding

The GVHD scoring and tumor size measuring were performed in a blinded manner and the codes were broken only after data collection was completed.
For other experimental procedures, blinding was not maintained to ensure accurate sample handling and prevent mislabeling between genotypes or treatment groups. However, to minimize bias, these data were analyzed using objective, quantitative methods rather than qualitative assessment.

Behavioural & social sciences study design

All studies must disclose on these points even when the disclosure is negative.

Study description

Briefly describe the study type including whether data are quantitative, qualitative, or mixed-methods (e.g. qualitative cross-sectional, quantitative experimental, mixed-methods case study).

Research sample

State the research sample (e.g. Harvard university undergraduates, villagers in rural India) and provide relevant demographic information (e.g. age, sex) and indicate whether the sample is representative. Provide a rationale for the study sample chosen. For studies involving existing datasets, please describe the dataset and source.

Sampling strategy

Describe the sampling procedure (e.g. random, snowball, stratified, convenience). Describe the statistical methods that were used to predetermine sample size OR if no sample-size calculation was performed, describe how sample sizes were chosen and provide a rationale for why these sample sizes are sufficient. For qualitative data, please indicate whether data saturation was considered, and what criteria were used to decide that no further sampling was needed.

Data collection

Provide details about the data collection procedure, including the instruments or devices used to record the data (e.g. pen and paper, computer, eye tracker, video or audio equipment) whether anyone was present besides the participant(s) and the researcher, and whether the researcher was blind to experimental condition and/or the study hypothesis during data collection.

Timing

Indicate the start and stop dates of data collection. If there is a gap between collection periods, state the dates for each sample cohort.

Data exclusions

If no data were excluded from the analyses, state so OR if data were excluded, provide the exact number of exclusions and the rationale behind them, indicating whether exclusion criteria were pre-established.

Non-participation

State how many participants dropped out/declined participation and the reason(s) given OR provide response rate OR state that no participants dropped out/declined participation.

Randomization

If participants were not allocated into experimental groups, state so OR describe how participants were allocated to groups, and if allocation was not random, describe how covariates were controlled.

Ecological, evolutionary & environmental sciences study design

All studies must disclose on these points even when the disclosure is negative.

Study description

Briefly describe the study. For quantitative data include treatment factors and interactions, design structure (e.g. factorial, nested, hierarchical), nature and number of experimental units and replicates.

Research sample

Describe the research sample (e.g. a group of tagged Passer domesticus, all Stenocereus thurberi within Organ Pipe Cactus National Monument), and provide a rationale for the sample choice. When relevant, describe the organism taxa, source, sex, age range and any manipulations. State what population the sample is meant to represent when applicable. For studies involving existing datasets, describe the data and its source.

Sampling strategy

Note the sampling procedure. Describe the statistical methods that were used to predetermine sample size OR if no sample-size calculation was performed, describe how sample sizes were chosen and provide a rationale for why these sample sizes are sufficient.

Data collection

Describe the data collection procedure, including who recorded the data and how.

Timing and spatial scale

Indicate the start and stop dates of data collection, noting the frequency and periodicity of sampling and providing a rationale for these choices. If there is a gap between collection periods, state the dates for each sample cohort. Specify the spatial scale from which the data are taken

Data exclusions

If no data were excluded from the analyses, state so OR if data were excluded, describe the exclusions and the rationale behind them, indicating whether exclusion criteria were pre-established.

Reproducibility

Describe the measures taken to verify the reproducibility of experimental findings. For each experiment, note whether any attempts to repeat the experiment failed OR state that all attempts to repeat the experiment were successful.

Randomization	*Describe how samples/organisms/participants were allocated into groups. If allocation was not random, describe how covariates were controlled. If this is not relevant to your study, explain why.*
Blinding	*Describe the extent of blinding used during data acquisition and analysis. If blinding was not possible, describe why OR explain why blinding was not relevant to your study.*

Did the study involve field work? ☐ Yes ☐ No

Field work, collection and transport

Field conditions	*Describe the study conditions for field work, providing relevant parameters (e.g. temperature, rainfall).*
Location	*State the location of the sampling or experiment, providing relevant parameters (e.g. latitude and longitude, elevation, water depth).*
Access & import/export	*Describe the efforts you have made to access habitats and to collect and import/export your samples in a responsible manner and in compliance with local, national and international laws, noting any permits that were obtained (give the name of the issuing authority, the date of issue, and any identifying information).*
Disturbance	*Describe any disturbance caused by the study and how it was minimized.*

Reporting for specific materials, systems and methods

We require information from authors about some types of materials, experimental systems and methods used in many studies. Here, indicate whether each material, system or method listed is relevant to your study. If you are not sure if a list item applies to your research, read the appropriate section before selecting a response.

Materials & experimental systems

n/a	Involved in the study	
☐	☒	Antibodies
☐	☒	Eukaryotic cell lines
☒	☐	Palaeontology and archaeology
☐	☒	Animals and other organisms
☒	☐	Clinical data
☒	☐	Dual use research of concern
☒	☐	Plants

Methods

n/a	Involved in the study	
☒	☐	ChIP-seq
☐	☒	Flow cytometry
☒	☐	MRI-based neuroimaging

Antibodies

Antibodies used	The following antibodies were used for FACS analysis: anti-CD80-FITC (16-10A1, 1:200, Biolegend#104706), anti-CD40-PE (FGK45, 1:500, Biolegend#157506), anti-NK1.1-PE (PK136, 1:100, Biolegend#108708), anti-CD4-violetFluor 450 (GK1.5, 1:200, Tonbo#75-0041), anti-CD8-violetFluor 500 (2.43, 1:200, Tonbo#85-1886), anti-CD27-Pacific Blue (LG.3A10, 1:500, Biolegend#124218), anti-CD44-PerCP-Cy5.5 (IM7, 1:200, Biolegend#103032), anti-CD45-PE (30-F11, 1:200, Biolegend#103106),anti-CD62L-PE (MEL-14, 1:200, Biolegend#104408), anti-CD69-violetFluor 450 (H1.2F3, 1:100, Cytek Biosciences#75-0691), anti-Foxp3-PerCP-eFluor710 (FJK-16s, 1:200, eBioscience#46-5773-82), anti-GzmB-FITC (GB11, 1:100, Biolegend#515403), anti-Klrg1-FITC (2F1, 1:500, Biolegend#138410), anti-Ki67-Pacific Blue (16A8, 1:100, Biolegend#652422), anti-Rorgt-PE (AFKJS-9, 1:200, eBioscience#12-6988-82), anti-Tbet-Pe-Cy7 (4B10, 1:200, Biolegend#644823), anti-IFNg-APC (XMG1.2, 1:100, Biolegend#505810), anti-TNFa-BV711 (MP6-XT22, 1:100, Biolegend#506349), anti-IL1b-eFluor450 (NJTEN3, 1:100, eBioscience#48-7114-82), anti-IL10-PE (JES5-16E3, 1:100, Biolegend#505008), anti-CD326-FITC (G8.8, 1:200, Biolegend#118208), anti-CD326-BV711 (G8.8, 1:200, Biolegend#118233), anti-Annexin V-APC (1:20, Biolegend#640920), anti-H2kb-BV421 (AF6-88.5, 1:100, Biolegend#116525), anti-IA/IE-APC (M5/114.15.2, 1:100, Biolegend#107614)<br><br>The following antibodies were used for Immunoblot analysis: anti-IRF1 (1:1000, CST#8478), anti-ACSL4 (1:3000, Abcam#ab155282), anti-b-actin (1:1000, CST#5125)
Validation	All antibodies were validated by the manufacturers for use in the requested applications as indicated on their websites.

Eukaryotic cell lines

Policy information about cell lines and Sex and Gender in Research

Cell line source(s)	B16-F0 cells (B16, #CRL-6322) were purchased from ATCC.

Authentication

Cell line was authenticated by ATCC and was used at low passage numbers.

Mycoplasma contamination

Cell line was negative for mycoplasma contamination in our laboratory.

Commonly misidentified lines
(See ICLAC register)

No commonly misidentified cell lines were used in this study

Animals and other research organisms

Policy information about studies involving animals; ARRIVE guidelines recommended for reporting animal research, and Sex and Gender in Research

Laboratory animals

C57BL/6 mice were purchased from Charles River. B6.Cg-Rag1tm1MomTyrp1B-wTg(Tcra,Tcrb)9Rest/J (#008684), B6(C)-H2-Ab1bm12/KhEgJ (#001162), B6.129P2-B2mtm1Unc/DcrJ (#002087), B6(Cg)-B2mtm1c(EUCOMM)Hmgu/J (#034858), B6.Cg-Tg(Vil1-cre)997Gum/J (#004586), B6;129S2-Tap1tm1Arp/J (#002458), STOCK Gpx4tm1.1Qra/J (#027964), B6.Cg-Tg(Vil1-cre/ERT2)23Syr/J (#020282) mice were purchased from The Jackson Laboratory (Bar Harbor, ME). ⍰2m floxed mice were bred with VilCre mice to generate B2mfl/flVilCre+ mice. ⍰2mfl/fl VilCre+ mice are referred to as B2mΔIEC mice. Gpx4fl/fl mice were bred with Vil1-cre/ERT2 mice to generate a conditional knockout model. Mice receiving tamoxifen injections were designated as Gpx4ΔIEC, while corn oil-treated Gpx4fl/fl littermates served as WT controls. The age of mice used for experiments ranged between 7 and 12 weeks. Mice were housed in a specific-pathogen-free (SPF) facility under a 12:12 h light/dark cycle. Ambient temperature was maintained at 20–24°C with a relative humidity of 45–65%. Mice had ad libitum access to standard chow and water throughout the study.

Wild animals

No wild animals were used in this study.

Reporting on sex

Both male and female mice were used in this study. For the majority of in vivo experiments, including the GVHD model, female mice aged 7–12 weeks were used to ensure cohort stability and minimize stress-induced variability caused by aggressive behavior (fighting) typically observed in male cohorts. However, key in vivo findings were independently reproduced in age-matched male mice to ensure the biological relevance and reproducibility of the results across both sexes. No sex-specific differences in the observed phenotypes were identified.

Field-collected samples

No field-collected animals were used in this study.

Ethics oversight

All mice were kept under specific pathogen-free conditions and cared for according to regulations reviewed and approved by the University of Michigan Committee on the Use and Care of Animals (PRO00009494) and Institutional Animal Care and Use Committee of Baylor College of Medicine (AN-8909).

Note that full information on the approval of the study protocol must also be provided in the manuscript.

Plants

Seed stocks

Report on the source of all seed stocks or other plant material used. If applicable, state the seed stock centre and catalogue number. If plant specimens were collected from the field, describe the collection location, date and sampling procedures.

Novel plant genotypes

Describe the methods by which all novel plant genotypes were produced. This includes those generated by transgenic approaches, gene editing, chemical/radiation-based mutagenesis and hybridization. For transgenic lines, describe the transformation method, the number of independent lines analyzed and the generation upon which experiments were performed. For gene-edited lines, describe the editor used, the endogenous sequence targeted for editing, the targeting guide RNA sequence (if applicable) and how the editor was applied.

Authentication

Describe any authentication procedures for each seed stock used or novel genotype generated. Describe any experiments used to assess the effect of a mutation and, where applicable, how potential secondary effects (e.g. second site T-DNA insertions, mosiacism, off-target gene editing) were examined.

Flow Cytometry

Plots

Confirm that:

☒ The axis labels state the marker and fluorochrome used (e.g. CD4-FITC).

☒ The axis scales are clearly visible. Include numbers along axes only for bottom left plot of group (a 'group' is an analysis of identical markers).

☒ All plots are contour plots with outliers or pseudocolor plots.

☒ A numerical value for number of cells or percentage (with statistics) is provided.

Methodology

Sample preparation

Briefly, to analyze splenocytes or intestinal epithelial cells or B16 cell lines, cells were suspended in FACS buffer (2% fetal bovine serum in PBS) and stained with conjugated antibodies.

Instrument	Cells were run on an Attune NxT flow cytometer or a Cytek Northern Lights flow cytometer.
Software	SpectroFlo v3.3.0 (Cytek Biosciences) was used to collect the data. FlowJo v10.10.0 was used for analyzing data.
Cell population abundance	No sorting was performed.
Gating strategy	A consistent gating strategy was applied to all samples to ensure reproducibility. Initial cells were identified using FSC-A/SSC-A to exclude debris. Doublet exclusion was then performed by gating on FSC-A/FSC-H to ensure single-cell analysis. Dead cells were subsequently excluded using Live/Dead NIR fixable viability dye. From the live cell population, intestinal epithelial cells (IECs) were identified as CD326+ cells.

☒ Tick this box to confirm that a figure exemplifying the gating strategy is provided in the Supplementary Information.
