# Supplementary figures and images for: MHC class I on target cells regulates CD4+ T cell-mediated immunity
Source: Nat Immunol. 2026 Mar 24;27(5):1000–12. doi: 10.1038/s41590-026-02480-z (PMC13132726; doi:10.1038/s41590-026-02480-z)

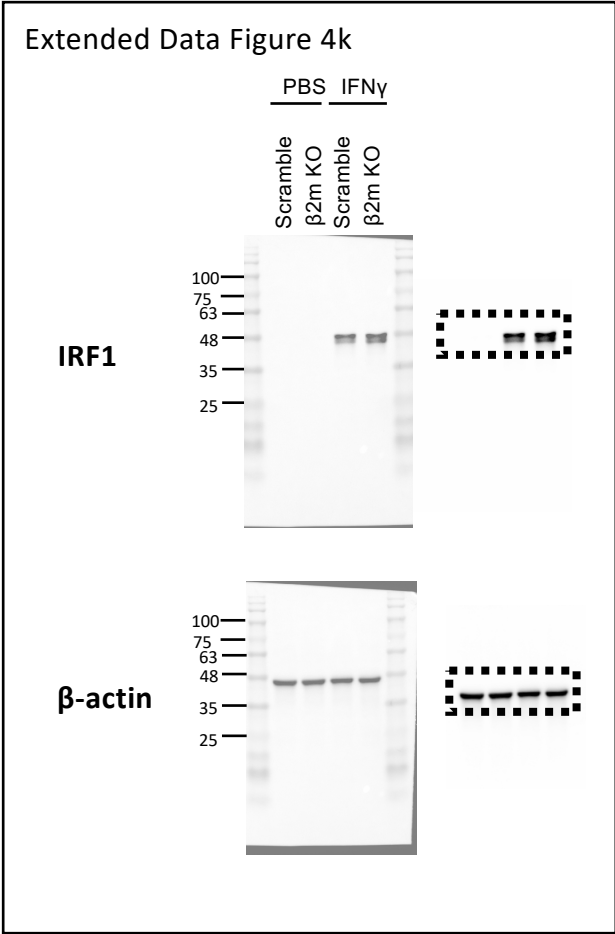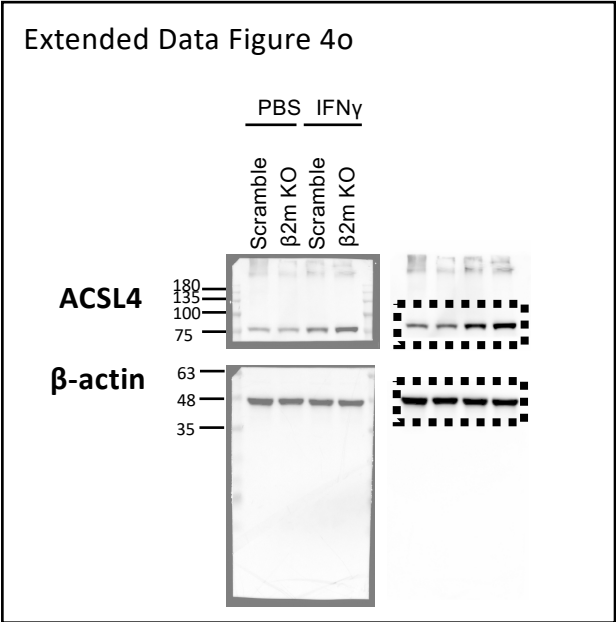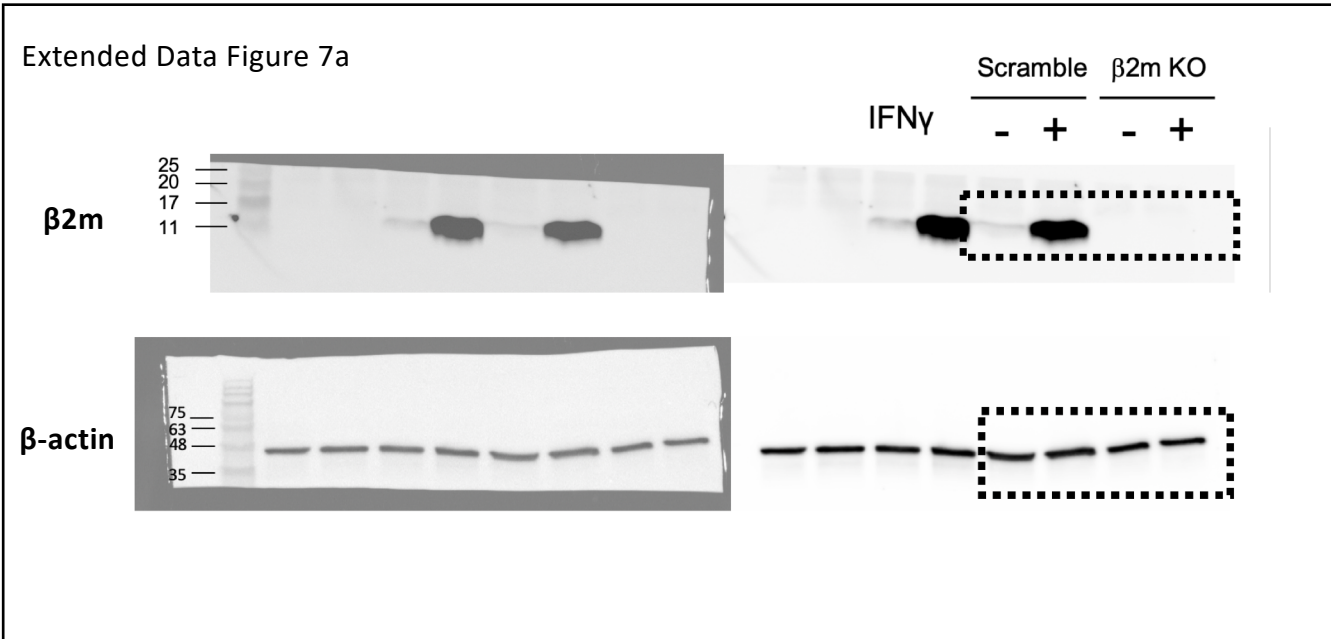

Supplement: Supplementary file 3 — Unprocessed western blots. [file 41590_2026_2480_MOESM3_ESM.pdf]
